# Oncosuppressive miRNAs loaded in lipid nanoparticles potentiate targeted therapies in BRAF-mutant melanoma by inhibiting core escape pathways of resistance

**DOI:** 10.1038/s41388-022-02547-9

**Published:** 2022-11-23

**Authors:** Luigi Fattore, Giordana Cafaro, Marta Di Martile, Virginia Campani, Andrea Sacconi, Domenico Liguoro, Emanuele Marra, Sara Bruschini, Daniela Stoppoloni, Roberto Cirombella, Francesca De Nicola, Matteo Pallocca, Ciro F. Ruggiero, Vittorio Castaldo, Angiolina Catizone, Donatella Del Bufalo, Giuseppe Viglietto, Andrea Vecchione, Giovanni Blandino, Luigi Aurisicchio, Maurizio Fanciulli, Paolo A. Ascierto, Giuseppe De Rosa, Rita Mancini, Gennaro Ciliberto

**Affiliations:** 1 SAFU Laboratory, Department of Research, Advanced Diagnostics and Technological Innovation, Translational Research Area, IRCCS Regina Elena National Cancer Institute, 00144 Rome, Italy; 2 Preclinical Models and New Therapeutic Agents Unit, IRCCS Regina Elena National Cancer Institute, Via Elio Chianesi 53, 00144 Rome, Italy; 3 Dipartimento Di Farmacia, Università Degli Studi Di Napoli Federico II, via D. Montesano 49, 80131 Naples, Italy; 4 Clinical Trial Center, Biostatistics and Bioinformatics Unit, IRCCS Regina Elena National Cancer Institute, 00144 Rome, Italy; 5 Department of Clinical and Molecular Medicine, Sapienza University of Rome, 00161 Rome, Italy; 6 Faculty of Medicine and Psychology, Department Clinical and Molecular Medicine, Sant’Andrea Hospital-Sapienza University of Rome, 00118 Rome, Italy; 7 Takis s.r.l., 00128 Rome, Italy; 8 Unit of Melanoma, Cancer Immunotherapy and Development Therapeutics, Istituto Nazionale Tumori IRCCS Fondazione Pascale, 80131 Naples, Italy; 9 Department of Anatomy, Histology, Forensic- Medicine and Orthopedics, Sapienza University of Rome, 00161 Rome, Italy; 10 Department of Experimental and Clinical Medicine, Magna Graecia University of Catanzaro, 88100 Catanzaro, Italy; 11 Oncogenomic and Epigenetic Unit, IRCCS Regina Elena National Cancer Institute, 00144 Rome, Italy; 12 Scientific Directorate, IRCSS Regina Elena National Cancer Institute, 00144 Rome, Italy

**Keywords:** Melanoma, Cell growth

## Abstract

BRAF-mutated melanoma relapsing after targeted therapies is an aggressive disease with unmet clinical need. Hence the need to identify novel combination therapies able to overcome drug resistance. miRNAs have emerged as orchestrators of non-genetic mechanisms adopted by melanoma cells to challenge therapies. In this context we previously identified a subset of oncosuppressor miRNAs downregulated in drug-resistant melanomas. Here we demonstrate that lipid nanoparticles co-encapsulating two of them, miR-199-5p and miR-204-5p, inhibit tumor growth both in vitro and in vivo in combination with target therapy and block the development of drug resistance. Mechanistically they act by directly reducing melanoma cell growth and also indirectly by hampering the recruitment and reprogramming of pro-tumoral macrophages. Molecularly, we demonstrate that the effects on macrophages are mediated by the dysregulation of a newly identified miR-204-5p-miR-199b-5p/CCL5 axis. Finally, we unveiled that M2 macrophages programs are molecular signatures of resistance and predict response to therapy in patients. Overall, these findings have strong translational implications to propose new combination therapies making use of RNA therapeutics for metastatic melanoma patients.

## Introduction

Acquired resistance to target therapies remains a daunting issue [1]. BRAF-mutated, metastatic melanoma is a paradigmatic example of a disease where the benefit of target therapy is curtailed by the development of drug resistance. In this tumor setting, inhibitors of mitogen-activated protein kinases BRAF and MEK (MAPKi) elicit prompt responses in most patients. However, therapeutic efficacy is not durable and the median duration of response is of only about one year [2, 3]. To further aggravate this condition, acquired resistance to target therapy is also accompanied by cross-resistance to immunotherapy with Checkpoint Inhibitors (ICI) [4], which has been attributed to the development of an immunosuppressive tumor microenvironment (TME) [5]. Indeed, patients relapsing after treatment with MAPKi have a lower response rate to immunotherapy as compared to treatment-naive patients [6–8]. This evidence thwarts the implementation of the sequential use of targeted therapy followed by ICI, which had been originally proposed as an opportunity to prolong disease control. From a mechanistic point of view, cross-resistance has mainly been attributed to the reactivation of the MAPK pathway, which rewires a series of transcriptomic changes leading to immune evasion and exhaustion [4, 9]. These lines of evidence depict a cancer cell-centric model to explain cross-resistance between the two types of therapies.

Several studies have unveiled the involvement of genetic and non genetic mechanisms at the root of drug resistance [10–17]. In the last few years, our group focused on non genetic mechanisms adopted by melanoma cells to survive after MAPKi and to develop drug resistance [18–20]. In this context, we helped discovering the pivotal role of microRNAs (miRNAs) [21–23]. We initially identified oncosuppressor miR-579-3p as a novel master regulator of MAPKi resistance in BRAF-mutant melanomas [24]. Subsequently, we uncovered the involvement of a large number of miRNAs acting either as facilitators (i.e. oncomiRs) or antagonists of resistance (i.e. tumor suppressive miRNAs) through whole miRnome profiling of melanoma cells undergoing development of resistance to targeted therapies [25]. Among the most significantly deregulated candidates, we further characterized the biological activity of two oncosuppressors, namely miR-204-5p and miR-199b-5p and two oncomiRs, i.e. miR-4443 and miR-4488 [25]. By exploiting a combination of assays using miRNA mimics or antagonists, we demonstrated that these miRNAs modulate the establishment of drug resistance in vitro by affecting the efficacy of MAPKi. Given that the most promising results have been obtained through re-establishing the expression of the oncosuppressors miR-204-5p and miR-199b-5p, we went on to further develop them as therapeutics. The most promising approach for exploiting miRNAs as therapy forecasts their encapsulation in lipid nanoparticles (LNPs) to circumvent the main drawbacks of delivering naked RNAs, i.e. poor cellular uptake, off-target activity and nuclease degradation. Along this line, we recently reported the initial biophysical and biochemical characterization in vitro of LNPs carrying both miR-204-5p and miR-199-5p [26].

In this paper, we go further to assess the therapeutic potential of miR-204-5p/miR-199-5p encapsulating LNPs as well to unravel their mechanism of action. We provide experimental evidence that the systemic delivery of LNPs carrying oncosuppressor miRNAs strongly potentiates the antitumor efficacy of MAPKi therapy and blunts the development of drug resistance in BRAF-mutant melanoma xenografts. Most importantly, we present evidence about the capability of miRNA-loaded LNPs to hamper the development of an immune-suppressive TME instructed by drug resistant melanoma cells in vitro and in vivo by acting on the key extracellular factors involved. Starting from transcriptomic and bioinformatics analyses followed by experimental validation in human biopsies and in melanoma cells, we identify the core escape pathways of MAPKi resistance governed by miR-204-5p and miR-199b-5p which involve the alteration of several pro-angiogenic and pro-inflammatory cues and the recruitment and reprogramming of tumor associated pro-tumoral macrophages (TAMs), which can be kept under check by miRNA-delivered LNPs.

Taken together these results support the transition of new combination therapies to clinical trials that utilize RNA therapy for metastatic melanoma.

## Results

### In vivo delivery of oncosuppressor miRNAs miR-204-5p and miR-199-5p by LNPs strongly potentiates MAPKi therapy and blunts the development of drug resistance in BRAF mutant melanomas

We have recently reported about the initial biophysical and biochemical characterization in vitro of LNPs carrying both miR-204-5p and miR-199-5p (LNP-miRs) and showed that they are able to reduce BRAF mutant melanoma cell growth alone and in combination with MAPKi, both in drug sensitive and drug resistant melanoma cells in vitro [26].

Here, we sought to expand these findings in preclinical mouse models. No previous studies have tested the capability of LNP-miRs to reduce melanoma cell growth in vivo neither alone nor in combination with targeted therapy. For this reason, we first carried out preliminary set up studies to gain insights on the LNP dose and the time of treatments capable of achieving overexpression of miR-204-5p and miR-199b-5p coupled with the inhibition of their target genes in tumors. To this purpose, we took advantage of xenograft models of A375 melanoma cells treated with 20 μg or 40 μg of LNPs loaded with scrambled miRNA sequences (LNP-Scr) or LNP-miRs. Mice were sacrificed at different time points (i.e. 24, 48, 72 and 96 h) to analyze RNA extracted from tumors (see experimental design in Fig. S1A). Furthermore, tumor volumes were also measured. First of all, we observed that 40 μg of LNP-miRs was the only dosage able to reduce short term small tumor masses as compared to LNP-Scr treated mice (Fig. S1B). Also, at 72 h post-injection, we observed the highest level of overexpression of both miRNAs coupled with the highest degree of reduction of their mRNA targets within tumor samples (Fig. S1C, D).

**Fig. 1:**
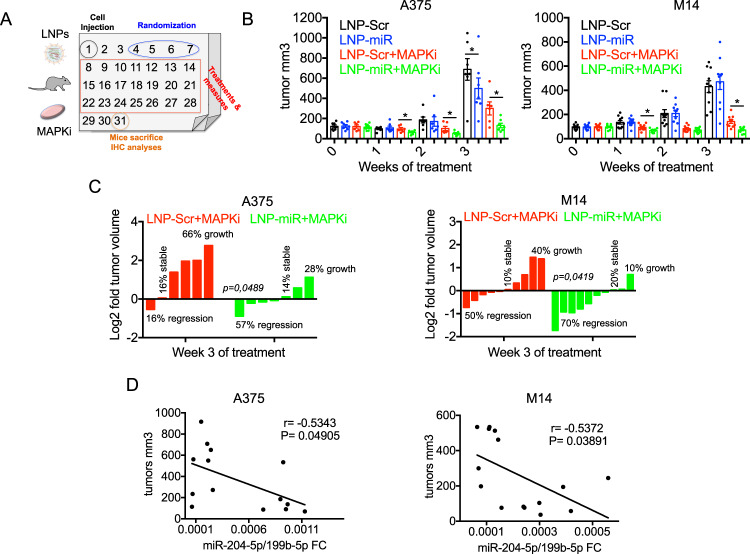
LNP-miRs potentiate MAPKi therapy in xenograft melanoma models. **A** Schematic workflow of the in vivo experiments performed with A375 or M14 melanoma cells treated with LNPs and/or MAPKi. **B** Bar plots showing the measures of the tumor volumes relative to the mice injected with A375 (*n* = 7) or M14 (*n* = 10) and treated with LNP-Scr, LNP-miRs or their combination with MAPKi. LNPs were injected via tail vein at the dose of 40 μg every 72 h, Dabrafenib+Trametinib (5days/week o.g) were used at the dose of 10 mg/kg+0.5 mg/kg, respectively for M14 cells and 5 mg/kg + 0.1 mg/kg, respectively for A375 cells. Treatments lasted for four weeks and tumor volumes were measured with a caliper. **C** The percentage of tumor shrinkage calculated respect to the initial volumes in mice injected with A375 (left panel) or M14 (right panel) belonging to the groups receiving LNP-Scr+MAPKi or LNP-miRs+MAPKi. **D** Spearman correlation between the tumor volumes and miR-204-5p/miR-199b-5p levels measured by qRT-PCR in A375 and M14 derived tumors. *p < 0.05; **p < 0.01; and ***p < 0.001. All in vivo results are represented as the mean (*n* = 7 for A375; *n* = 10 for M14) ± SEM.

Based on these results, we chose a 40 μg dose of LNPs administered every 72 h to study the efficacy of long-term treatment of LNP-miRs with MAPKi in mouse models injected with A375 (*n* = 7) or M14 (*n* = 10) melanoma cell lines. Treatments were performed for three weeks and tumor volumes measured once a week (Fig. 1A). Furthermore, we also measured mouse weight as a parameter to monitor animal suffering in response to the triple regimen (LNPs + BRAFi+MEKi). Of note, we did not observe any significant weight loss in mice as compared to the beginning of the study in both the models thus suggesting that the treatments were well tolerated by animals (Fig. S2A). LNP-miRs were able to strongly potentiate the inhibitory effects of target therapies keeping the tumor regrowth under control for a longer time as compared to LNP-Scr+MAPKi groups (Fig. 1B). It is worth mentioning that we observed a significant inhibition of A375 tumor volumes but not of M14 derived tumors also in the group treated with the sole LNP-miRs as compared to LNP-Scr treated mice. This may be due to the different efficiency in the intracellular uptake of miRNAs following LNPs’ treatments between the two cellular models as our in vitro data suggest [26]. To confirm this hypothesis in xenograft models, we measured the expression levels of miR-204-5p and miR-199b-5p by qRT-PCR in LNP-miRs treated mice. In line with expectations, we observed that the uptake of both miRs is significantly higher in A375-derived tumors as compared to M14-derived tumors (Fig. S2B).

Furthermore, by focusing on the groups LNP-Scr+MAPKi vs. LNP-miRs+MAPKi, we divided mice according to therapy response based on the percentage of tumor shrinkage compared to their initial volumes (Fig. 1C). Strikingly, we observed that LNP-miRs were able to significantly increase the proportion of mice responding to target therapies in both A375 and M14. In particular, in M14, we observed 50% regression, 10% stability and 40% tumor regrowth in the LNP-Scr+MAPKi group whereas 70% regression, 20% stability and 10% tumor regrowth in the LNP-miRs+MAPKi group. In A375 we observed 16% regression, 16% stability and 66% tumor regrowth in the LNP-Scr+MAPKi group whereas 57% regression, 14% stability and 28% tumor regrowth was observed in the LNP-miRs+MAPKi group.

Tumor masses were subjected to qRT-PCR, Western blot and immunohistochemistry (IHC). The level of expression of miR-204-5p and miR-199b-5p showed a negative Spearman correlation with tumor volumes both in A375 and M14 mouse models (Fig. 1D) according to the oncosuppressor potential of these miRNAs. Moreover, Western blot analyses showed a reduction of pERK activation in tumors treated with LNP-miRs+MAPKi as compared to the other groups (i.e. LNP-Scr, LNP-miRs or LNP-Scr+MAPKi) (Fig. S2C). Finally, tumor masses were further analyzed by IHC to determine: 1) cell proliferation using the ki67 marker, 2) percentage of necrosis and 3) neo-vessel formation through the endothelial cell marker CD31. A375 derived tumors showed a reduction of CD31 positive vessels in LNP-miRs+MAPKi vs. LNP-Scr+MAPKi groups but not in the percentage of necrosis and ki67 positive cells (Fig. S2D).

In contrast, M14 derived tumors showed a reduction of ki67 positive cells and an increase in percentage of necrosis in LNP-miRs+MAPKi vs. LNP-Scr+MAPKi groups but no differences in CD31 positive vessels (Fig. S2E). In summary, our results show for the first time that the in vivo systemic delivery of LNPs encapsulating oncosuppressor miRNAs potentiate target therapies for BRAF-mutant melanomas and significantly delay the emergence of drug resistance.

### miR-204-5p and miR-199b-5p control core escape pathways involved in MAPKi resistance

**Fig. 2:**
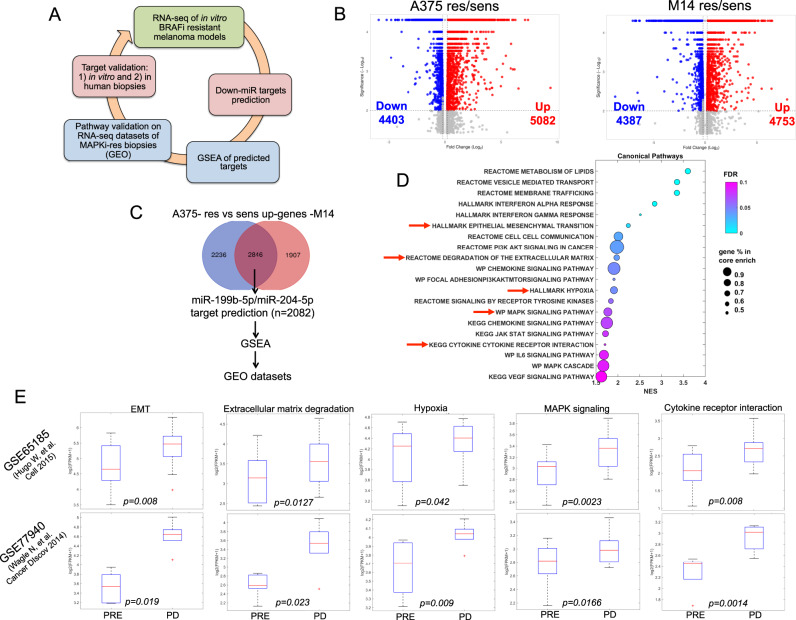
Core escape pathways of MAPKi resistance in melanoma are controlled by miR-204-5p and miR-199b-5p. **A** Schematic illustration of the experimental approach to identify the molecular pathways/targets regulated by oncosuppressive miRNAs impacting MAPKi resistance. **B** Volcano plots of differentially expressed genes (DEGs) coming from bulk RNA sequencing (RNA-seq) of A375 and M14 BRAFi-resistant cells vs. sensitive counterparts (Log2Fold Change>0.15 and adjusted *p* value < 0.05). **C** Venn Diagram of the commonly up-regulated genes of res/sens cells potentially targeted by miR-204-5p and/or miR-199b-5p. **D** Bubble plot of Gene Set Enrichment Analysis (GSEA) showing 20 enriched gene expression signatures of BRAFi resistant cells. **E** Box plots representing 5 pathways enriched in biopsies of therapy resistant patients (datasets of bulk RNA-seq numbers GSE65185 and GSE77940): epithelial to mesenchymal transition (EMT), degradation of the extracellular matrix, hypoxia, MAPK signaling pathway and cytokine-cytokine receptor interaction. GSEA was run in preranked mode using classic as metric and 1000 permutations (FDR < 0.1, *p* value < 0.05, logfold>0.3).

We next decided to investigate in detail the molecular pathways affected by the two oncosuppressor miRNAs (briefly Down-miRs) which are both downregulated in MAPKi-resistant melanoma cells [25]. To this purpose, we conceived the experimental approach depicted in Fig. 2A.

A375 and M14, rendered resistant to a BRAFi *vs*. their sensitive counterparts were subjected to bulk RNA sequencing (RNA-seq). Differential expression analysis (DEA) revealed a massive rewiring of the transcriptome in both drug resistant cellular models (see volcano plots in Fig. 2B). In particular, we found 9485 differentially expressed genes (DEGs) in A375-res/sens (5082 up-regulated and 4403 downregulated) and 9140 DEGs in M14-res/sens (4753 up-regulated and 4387 downregulated). The complete lists of the deregulated genes (Log2Fold Change| > 0.15 and adjusted *p* value < 0.05) together with the biological function of DEGs assessed by Gene Ontology (GO) analyses are available as Supplementary Data 1. Besides transcriptomic analyses, we also evaluated the occurrence of genomic alterations in A375 resistant cells vs. sensitive counterparts by the Whole Exome Sequencing (WES). Our data showed that most genetic variants (about 90% of the total) are similar between A375-res/sens cells (see Venn diagrams in Fig. S3A). This trend is confirmed with germline and somatic-specific filters. Surprisingly, we did not find the occurrence of somatic driver mutations in genes involved in MAPK-reactivating mechanisms and PI3K–PTEN–AKT pathway up-regulation (Fig. S3B) [14]. These findings have been experimentally confirmed by measuring pERK and pAKT activation through Western blot analyses in both A375 and M14 res vs sens cells (Fig. S3C). The complete lists of the genomic variants are available in Supplementary Data 2. These data suggest that transcriptomic changes dominate over genomic alterations to drive BRAFi resistance at least in our model.

Moving forward, we decided to focus on the commonly up-regulated genes in A375 and M14 res/sens cells to identify the potential oncogenic drivers of resistance. This led to the identification of 2846 potential candidates. This list was filtered using the prediction tool miRwalk3, which helped us to identify the potential target genes of at least one microRNA between miR-204-5p and miR-199b-5p. Interestingly, we found that 2082 out of the 2846 up-regulated genes (about 73% of the total) are potential targets of the two Down-miRs (Fig. 2C). This suggests that miR-204-5p/miR-199b-5p are key regulators of the transcriptome of drug resistant melanoma cells. The complete list of genes is available in Supplementary Data 3A. We then subjected this list to the Gene Set Enrichment Analysis (GSEA) which allowed to identify 20 most relevant enriched gene expression signatures between drug resistant vs. sensitive cells shown as a bubble plot in Fig. 2D (the full lists are available as Supplementary Data 3B, C). To confirm the validity of this approach, we checked these gene signatures in two independent datasets of bulk RNA-seq from melanoma biopsies sequenced before (Pre) or after development of resistance (PD) to targeted therapies. These data are available in the Gene Expression Omnibus database under accession codes GSE65185 and GSE77940. Results demonstrate that 5 out of the 20 pathways identified are significantly enriched in biopsies from therapy resistant patients (Fig. 2E), namely: epithelial to mesenchymal transition (EMT), degradation of the extracellular matrix, hypoxia, MAPK signaling pathway and cytokine-cytokine receptor interaction (see red arrows in Fig. 2D). The full lists of the genes and the molecular pathways affected are reported in Supplementary Data 3D, E. It is important to point out that these core escape pathways had already been identified as being involved in non-genetic resistance to MAPKi in melanoma [4, 17, 27]. Hence we can conclude that the activation of these pathways is tightly linked to the downregulation of miR-204-5p and miR-199b-5p and, therefore, controlled by the expression of these two microRNAs.

### Pro-angiogenic and pro-inflammatory cues controlled by oncosuppressor miRNAs are hallmarks of MAPKi resistant melanomas

**Fig. 3:**
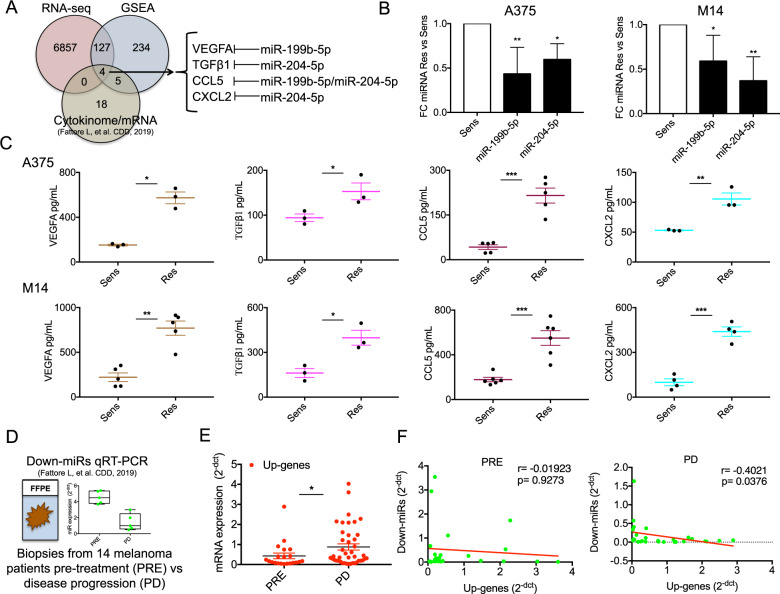
Pro-angiogenic and pro-inflammatory factors controlled by oncosuppressor miRNAs are distinguishing features of MAPKi-resistant melanomas. **A** Venn Diagram showing the intersected data obtained by RNA-seq, GSEA and cytokinome analyses for the identification of VEGFA, TGFβ1, CCL5 and CXCL2. **B** Quantification of miR-199b-5p and miR-204-5p by using qRT–PCR in A375 and M14 BRAFi-resistant cells vs. sensitive counterparts. **C** Elisa assays measuring VEGFA, TGFβ1, CCL5 and CXCL2 soluble levels in cell media (CM) deriving from A375 (upper panels) and M14 (lower panels). For these experiments cells have been serum starved for 24 h and then CM have been collected; results were determined by measuring absorbance at 450 nm into a microplate reader. **D** Representative results of miR-204-5p and miR-199b-5p expression from matched formalin-fixed paraffin-embedded (FFPE) melanoma samples before initiation of targeted therapy (Pre) and after disease progression (PD). **E** VEGFA, TGFβ1, CCL5 and CXCL2 mRNA expression levels (Up-genes) measured by qRT-PCR in PD vs. Pre-therapy samples (*n* = 14). **F** Spearman correlation calculated using qRT-PCR data of the two Down-miRs (miR-204-5p and miR-199b-5p) and the four Up-genes in PD (right panel) and in Pre-therapy (left panel) samples. *p < 0.05; **p < 0.01; and ***p < 0.001. qRT-PCR data are represented as the mean (*n* = 3) ±SD; Elisa results are expressed as mean of at least three independent experiments ± SEM.

In the previous section, we have shown that both in vitro melanoma models and human biopsies share commonly enriched gene signatures of acquired resistance to MAPKi potentially regulated by the couple of oncosuppressor miRNAs miR-204-5p/miR-199b-5p. We reasoned that the deregulated pathways identified by GSEA involve the expression of pro-angiogenic and pro-inflammatory factors. This idea was supported by our previous analysis of the cytokinome profile of MAPKi resistant *vs*. sensitive cells which revealed the overproduction and release of a wide repertoire of growth factors, cytokines and chemokines potentially able to reprogram a drug resistant tumor microenvironment [25]. Therefore, in order to identify the molecular targets of miR-204-5p and/or miR-199b-5p at play, we intersected data obtained by RNA-seq, GSEA and cytokinome analyses. Criteria for selection were: 1) up-regulation in profiling experiments (i.e. RNA-seq and/or cytokinome) in at least one cell culture resistant model, 2) presence in the list of the top gene signatures identified by GSEA (i.e. EMT, degradation of the extracellular matrix, hypoxia, MAPK signaling pathway and cytokine-cytokine receptor interaction) and 3) direct molecular targets of miR-204-5p and/or miR-199b-5p. This led to identifying the following four factors, i.e. VEGFA, TGFβ1, CCL5 and CXCL2 as the only candidates satisfying all the above mentioned criteria (Fig. 3A). The full lists of the genes used to plot Venn Diagram of Fig. 3A are available as Supplementary Data 4.

In order to validate these findings, we first confirmed the downregulation of the two oncosuppressor miRNAs in A375 and M14 res/sens cells through qRT-PCR (Fig. 3B). Afterwards, we measured the expression levels of the four candidates in the same in vitro resistant models. Given that VEGFA, TGFβ1, CCL5 and CXCL2 are released in cell media (CM) as soluble factors, we collected the supernatants from drug resistant A375 and M14 melanoma cells and from their sensitive counterparts. Results demonstrated that CM from resistant cells were strongly enriched of VEGFA, TGFβ1, CCL5 and CXCL2 in both the cell lines tested (Fig. 3C). These results were confirmed also in a cell line rendered double resistant to both BRAF and MEK inhibitors, i.e. A375DR (Fig. S5A, B). We then validated these findings in human biopsies. To this purpose, we analyzed total RNA extracted from 14 matched formalin-fixed paraffin-embedded (FFPE) melanoma samples before initiating targeted therapy (Pre) and after disease progression (PD). In these specimens, we already demonstrated the downregulation of both miR-204-5p and miR-199b-5p in relapsing lesions as compared to pre-therapy samples through qRT-PCR (representative results of [25] in Fig. 3D). Of note the higher levels of these miRNAs also correlate with a better survival of melanoma patients through interrogation of Skin Cutaneous Melanoma (SKCM) data from The Cancer Genome Atlas (TCGA) (*n* = 471) (Fig. S4). Moving forward, we evaluated the mRNA expression levels relative to VEGFA, TGFβ1, CCL5 and CXCL2 in these solid biopsies (from here simply Up-genes). Results (Fig. 3E) confirmed the up-regulation of these cytokines and chemokines in PD as compared to pre-therapy samples. Finally, we observed a significant negative Spearman correlation (*r* = −0.4021; *p* = 0.0376) between the two Down-miRs (i.e. miR-204-5p and miR-199b-5p) and the four Up-genes (i.e. VEGFA, TGFβ1, CCL5 and CXCL2) only in melanoma biopsies progressed after targeted therapies (PD samples) (Fig. 3F, right panel). Differently, in pre-therapy lesions we observed a similar trend without reaching statistical significance (*r* = −0.01923; *p* = 0.9273) (Fig. 3F, left panel).

These results support the notion that the development of drug resistance upsets the balance between the two miRNAs and their targets as compared to pre-therapy. Altogether, our data demonstrate that the aberrant production of the above mentioned pro-angiogenic and pro-inflammatory factors is correlated to the downregulation of miR-204-5p and miR-199b-5p in MAPKi-resistant melanomas.

### Oncosuppressor miRNAs delivered by lipid nanoparticles exert a dual inhibitory function on MAPKi resistant melanoma cells

**Fig. 4:**
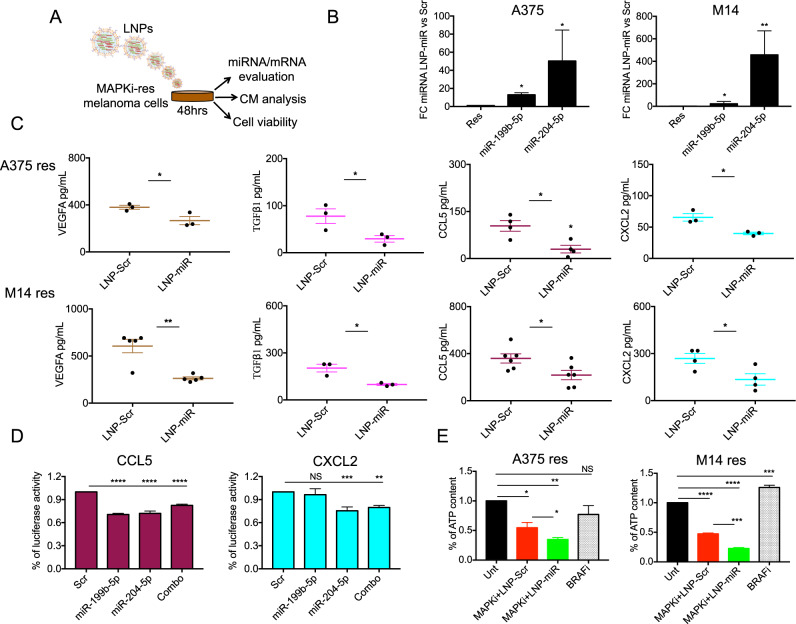
LNP-miRs reduce the levels of VEGFA, TGFβ1, CCL5 and CXCL2 and inhibit the proliferation of BRAFi resistant melanoma cells. **A** Schematic illustration of the biological assays carried out upon melanoma cells exposure to LNPs. **B** Quantification of miR-199b-5p and miR-204-5p by using qRT–PCR in A375 and M14 BRAFi-resistant cells upon 48 h of exposure to LNP-Scr or LNP-miRs. **C** Elisa assays measuring VEGFA, TGFβ1, CCL5 and CXCL2 soluble levels in CM coming from A375 res (upper panels) and M14 res (lower panels) cells upon LNPs’ exposure (48 h). For these experiments cells have been serum starved for 24 h and then CM have been collected; results were determined by measuring absorbance at 450 nm into a microplate reader. **D** Luciferase reporter assays of the constructs containing CCL5 and CXCL2 3’UTRs co-transfected with the indicated miRNAs alone or in combination for 48 h have been performed in HEK293 cells. CCL5 and CXCL2 reporter plasmids were transfected at 500 ng; pLX313-Renilla plasmid has been used to normalize results at 50 ng. **E** Cell viability evaluation by measuring ATP content in A375 and M14 BRAFi-resistant cells left untreated, treated with the sole Dabrafenib (BRAFi, 500 nM) or in combination with the MEKi, i.e. Trametinib at 10 nM (MAPKi) in the presence of LNP-Scr or LNP-miR (30 μg each). *p < 0.05; **p < 0.01; and ***p < 0.001. qRT-PCR data are represented as mean (*n* = 3) ± SD; Elisa, luciferase and cell viability results are expressed as the mean of at least three independent experiments ±SEM.

We have recently demonstrated that LNPs carrying miR-204-5p and miR-199b-5p mimics are able to inhibit melanoma cell growth both alone and in combination with MAPKi [26]. Here, we decided to test the ability of these nanoparticles to inhibit the expression of pro-angiogenic and pro-inflammatory factors shown in the previous section to be released by MAPKi resistant cells. To this purpose, we exposed A375 and M14 res cells for 48 h to LNP-Scr or LNP-miRs and carried out different biological assays (Fig. 4A). First, we evaluated the expression levels of miR-199b-5p and miR-204-5p in melanoma cells following LNP treatments by qRT-PCR. Results confirmed the intracellular delivery of both miRNAs and in line with our previous data, we observed that the increase of miR-204-5p occurs at higher levels compared to miR-199b-5p (Fig. 4B). We then collected CM from A375 and M14 res cells upon exposure to LNPs to measure the level of released VEGFA, TGFβ1, CCL5 and CXCL2. Elisa assays demonstrated that Down-miRs enhanced intracellular concentration was able to significantly reduce the levels of these factors (Fig. 4C). These results were confirmed also in A375DR cells (Fig. S5C, D).

It has already been reported that miR-204-5p is a negative regulator of TGFβ1 [28, 29] and that miR-199b-5p is able to downregulate VEGFA [30]. For the latter, we previously demonstrated that this miRNA inhibits the pro-angiogenic stimuli sustained by VEGF pathway aberrantly altered in MAPKi resistant melanoma cells [25]. As to CCL5 and CXCL2, no previous data reported their targeting by miR-204-5p and/or miR-199b-5p. According to bioinformatic predictions (see Fig. 3A), CCL5 may be targeted by both Down-miRs because its 3’UTR has two bindings sites that match miR-199b-5p/miR-204-5p seed regions. In contrast, CXCL2’s 3’UTR contains only the binding site relative to miR-204-5p seed region. To experimentally validate in silico predictions, we carried out luciferase reporter assays. Results demonstrated that the restored intracellular level of both Down-miRs led to a significant inhibition of luciferase activity in the constructs containing CCL5 3’UTR as compared to the negative control. As expected, in the case of CXCL2, luciferase signal was reduced only in miR-204-5p transfected cells (Fig. 4D). Altogether, these results confirmed the validity of bioinformatic predictions and allowed to uncover two new molecular targets of miR-204-5p and miR-199b-5p. Besides the direct effects on CCL5/CXCL2 3’UTRs it is possible that additional indirect regulatory networks orchestrated by miR-204-5p and miR-199b-5p are at play as already demonstrated in other cancer types [31, 32].

Finally, we tested the capability of LNP-miRs to reduce the proliferation of drug resistant A375 and M14 melanoma cells in the presence of Dabrafenib+Trametinib (i.e. MAPKi). To this aim, cells were tested for the presence of metabolically active and viable cells. Results (Fig. 4E) show that LNP-miRs were able to potentiate the growth inhibitory effects of MAPKi on BRAFi-resistant melanoma cells. We also performed the same combinatorial experiments in BRAF-mutant WM115 melanoma cells that are intrinsically resistant to targeted therapies [18, 33]. Results demonstrate that LNP-miRs are not able to synergize with MAPKi in this cellular model thus suggesting that in intrinsic resistance different mechanisms and probably different miRNAs are at play (Fig. S5E).

In summary, these data show that LNP-miRs are able to exert a dual function on MAPKi resistant melanoma cells: a) a direct growth inhibitory effect if administered alone [26] or in combination with inhibitors of the MAPK pathway; b) an indirect effect on the TME shaped by drug-resistant melanoma cells by reducing the production and release of multiple pro-angiogenic and pro-inflammatory factors.

### Oncosuppressor miRNAs delivered by LNPs impair the recruitment of pro-tumoral macrophages by MAPKi resistant melanoma cells in vitro and in vivo

**Fig. 5:**
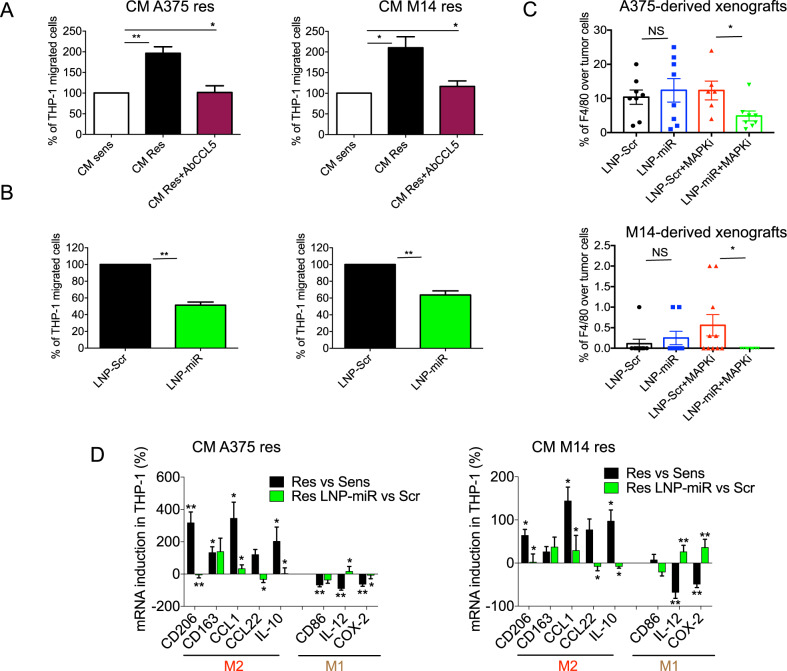
LNP-miRs reduce the capability of BRAFi-resistant cells to attract and reprogram M2 macrophages. **A** CM coming from A375 or M14 BRAFi sens/res melanoma cells used to induce the migration of THP-1 monocytes in Transwell chambers. **B** CM coming from resistant melanoma cells treated with LNP-Scr or LNP-miR and then used to induce the migration of THP-1 cells. For migration assays, 1×10^5^ THP-1 cells were plated in the upper chamber of Transwell and exposed to the indicated CM added in the lower chamber. After 3 h, cells remaining on the top side of the membrane were removed and migrating cells were fixed, stained and counted. **C** Quantification of immunohistochemistry (IHC) analyses evaluating macrophage infiltration within the A375 and M14 derived tumors using the F4/80 antibody. **D** qRT–PCR of the indicated M2 or M1 macrophages markers performed on THP-1 cells exposed to CM coming from res/sens melanoma cells (black bars) or CM coming from resistant cells treated with LNP-Scr or LNP-miR (green bars). For these experiments, THP-1 monocytes were differentiated in macrophages with 100 ng/mL phorbol-12-myristate- 13-acetate for 24 h and then exposed for another 24 h to cell media (CM) deriving from melanoma cells. *p < 0.05; **p < 0.01; and ***p < 0.001. qRT-PCR data are represented as the mean of at least three independent experiments ±SEM; migration results are expressed as the mean (*n* = 3) ±SD. All in vivo results are represented as the mean (*n* = 7 for A375; *n* = 10 for M14) ± SEM.

Next, we started to investigate which cellular components of the TME were potentially reprogrammed by drug-resistant melanoma cells through the release of their multiple soluble mediators. In this context, great importance has recently been attributed to the recruitment of myeloid cells, in particular TAMs [34]. Given that circulating monocytes are the major source of infiltrating macrophages in tumors [35], we first assessed the capability of drug resistant melanoma cells to recruit them. Therefore, through bioinformatic analyses we tested the correlation between upregulated genes and monocyte infiltration in SKCM data from TCGA. To this aim, we took advantage of the online software TIMER 2.0 [36], which allows an accurate estimation of immune-infiltrating levels based on bulk RNA-seq data (see methods’ section for details). By applying this approach, we observed that CCL5 significantly and positively correlated with monocyte infiltration in melanomas (Fig. S5). In contrast, VEGFA, TGFβ1 and CXCL2 were not significantly correlated with monocyte infiltration (Fig. S6). These data are in line with the known role of CCL5 as one of the main chemoattractants for monocytes within the tumor microenvironment [37]. Next, we went on to experimentally confirm in silico evaluations. To this aim, we tested the capability of THP-1 monocytes to migrate through transwell chambers in response to the chemotactic agents represented by CM derived from A375 and M14 melanoma cells. Results clearly showed that CM from resistant cells (CM Res) trigger the migration of THP-1 cells better than CM coming from sensitive counterparts (Fig. 5A). When we pre-incubated CM Res with an antibody directed against CCL5 (AbCCL5), their capability to trigger THP-1 cell migration was fully abrogated (Fig. 5A). We then exposed resistant melanoma cells to LNPs and collected CM 48 h upon treatment to perform migration experiments. Results demonstrated that LNP-miRs were able to inhibit the migration of THP-1 monocytes as compared to LNP-Scr treated cells (Fig. 5B). These data suggest that the inhibition of CCL5 by miR-204-5p/miR-199b-5p is sufficient to block the chemoattractant capability for monocytes of resistant CM.

We also decided to test whether macrophage recruitment also occur in vivo following MAPKi treatments analyzing the tumor masses collected from A375 and M14 melanoma xenografts (see Fig. 1). To this purpose, macrophage infiltration within the tumors was evaluated by IHC using the F4/80 antibody, a specific murine macrophage-related marker. As expected, mice belonging to LNP-miRs+MAPKi groups were characterized by tumors with the lowest percentage of F4/80 positive cells (Fig. 5C and representative images in Fig. S7). Interestingly, in M14 derived tumors macrophages were totally undetectable in LNP-miRs+MAPKi treated mice.

Finally, we decided to investigate how CM derived from drug resistant melanoma cells influences the biological fate of TAMs once recruited in the tumor niche. To answer this question, we induced the differentiation of THP-1 monocytes to macrophages and exposed them for 24 h to CM derived from resistant *vs*. sensitive A375 and M14 cells. Data show that CM from resistant cells was able to increase the levels of specific markers of pro-tumoral M2 macrophages (CD206, CD163, CCL1, CCL22 and IL-10) and, in contrast, lead to a significant reduction of anti-tumoral M1 markers, such as CD86, COX-2 and IL-12 (Fig. 5D, black bars). These results were observed both in A375 and M14 cells despite in the first ones at a higher magnitude. It is important to note that, when we treated resistant melanoma cells with LNP-miRs before collecting CM, we were able to reduce the M2 shift toward a M1 gene expression pattern (Fig. 5D, green bars). These results may be explained by the inhibition of VEGFA, TGFβ1, CCL5 and CXCL2 following oncosuppressor-miRs enforced expression in MAPKi-resistant cells (Fig. 4C and Fig. S8A, B). Of note, we have also tested the levels of other cytokines (i.e. IL6, TNF-α and IL1β) in THP-1 cells exposed to CM coming from melanoma cells and we have not observed any significant modulation (Fig. S8C). Finally, we revealed a positive correlation between the set of upregulated genes and M2 macrophage infiltration in melanomas based on TCGA data (Fig. S9).

Taken together these findings show that drug resistant melanoma cells attract and reprogram macrophages by inducing their pro-tumoral M2 polarization through the production of a series of soluble factors controlled by the two oncosuppressors miR-204-5p and miR-199b-5p. These macrophages can be re-educated toward an M1 anti-tumoral phenotype using LNPs carrying the same two miRNAs.

### The relative ratio of M2 vs. M1 is predictive of resistance to MAPKi therapy

**Fig. 6:**
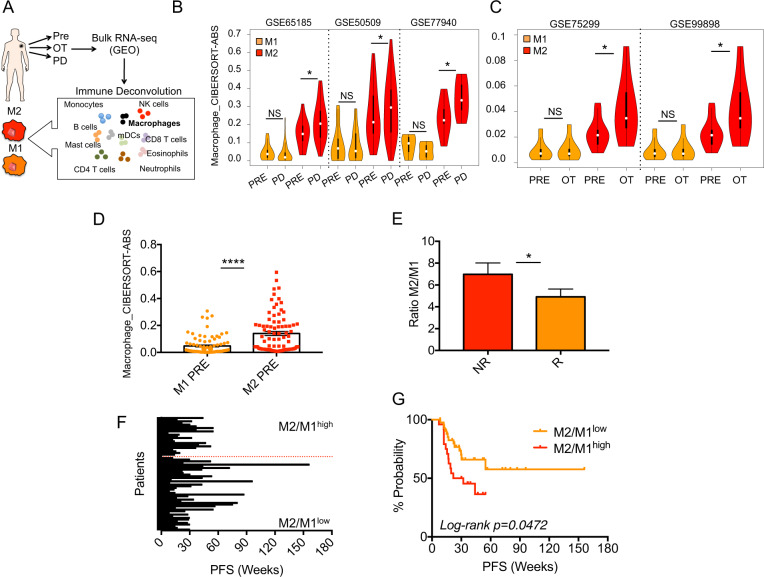
Pro-tumoral M2 macrophage transcriptional programs are molecular signatures of MAPKi resistance and predict therapy response in melanoma. **A** Schematic illustration of deconvolution analyses allowing to dissect the immune cell landscape of melanoma biopsies. To this aim bulk RNA-seq data available in the GEO database were subjected to the CIBERSORTx algorithm (TIMER2.0) to determine the proportion of 22 different immune cell types in each sample. Violin plots showing M1 and M2 gene signatures in relapsing (**B**) and on treatment (**C**) biopsies *vs*. treatment naïve lesions from the indicated datasets available the GEO database. **D** CIBERSORTx absolute (ABS) values of M2 and M1 transcriptional programs in basal biopsies of the aforementioned public datasets. **E** The ratio of M2/M1 gene programs from deconvolution analyses of RNA-seq data (five previous GEO datasets) distinguishing non responders (NR) from responders (R) melanoma patients. Bar plots (**F**) and Kaplan–Meier curves (**G**) showing M2/M1 ratio as predictor of Progression Free Survival (PFS) to MAPKi in melanoma patients. The hazard ratio, Cox models and the log-rank p values were evaluated to plot KM curves *p < 0.05; **p < 0.01; and ***p < 0.001. Log-rank *p* value < 0.05.

We attempted to confirm the clinical relevance of M2 macrophage infiltration in drug resistant tumors by using bulk RNA-seq data from melanoma patients available in the GEO database. In particular, we tested five datasets with the accession codes: GSE65185 (*n* = 62), GSE50509 (*n* = 59), GSE77940 (*n* = 12), GSE75299 (*n* = 19) and GSE99898 (*n* = 25) which all derived from melanoma biopsies sequenced before (PRE), during treatment (OT) and after resistance (PD) to targeted therapies. To assess M1 and M2 macrophage transcriptional programs, we subjected RNA-Seq data to deconvolution analyses through CIBERSORTx which allows to dissect the immune cell landscape of melanoma biopsies (Fig. 6A). Strikingly, results demonstrated that M2-related gene signatures were significantly higher in relapsing (Fig. 6B) and on treatment (Fig. 6C) biopsies vs. treatment naïve lesions. These data suggest that M2 infiltration levels increase early during MAPKi treatments and are further induced upon development of acquired resistance. CIBERSORTx absolute (ABS) values utilized to perform the differential deconvolution analyses are reported in Supplementary Data 5. The alteration of M2 signatures that we observed probably occurs together with that of other immune cells to reprogram an immunosuppressive TME. For example, it has been recently reported by exploiting our same deconvolution approach that the proportion of CD8 T-cells and NK-cells is lower in melanoma biopsies progressed from MAPKi as compared pre-treatment lesions [38].

Next, we asked whether macrophage infiltration levels before starting treatment may be a predictor of response to therapy. First of all, focusing on basal biopsies, we noticed that M2 transcriptional programs dominate over M1 programs (Fig. 6D) according to the malignant feature of these lesions. To estimate the predictive value of macrophage signatures, we calculated the ratio between M2/M1 gene programs starting from deconvolution analyses of RNA-seq data. We chose basal biopsies coming from the five aforementioned GEO datasets relative to patients for which clinical data were available (*n* = 67). Among them, we found 27 non responders (NR) and 40 responders (R) defined by the criteria outlined in the methods section. Data shown in Fig. 6E demonstrate that the M2/M1 ratio was significantly higher in NR vs. R patients, thus suggesting that an increased M2 infiltration before starting treatments is able to distinguish patients with a faster PD. To corroborate these findings, we used the M2/M1 ratio to construct receiver operating characteristic (ROC) curves in order to estimate the predictive value of this parameter as a predictor of response to MAPKi. Results measured as the Area Under Curve (AUC) showed that M2/M1 ratio yielded a significant AUC value of 0.616 (*p* value < 0.5, cutoff used: 4.74). Finally, we tested the M2/M1 ratio as a predictor of Progression Free Survival (PFS) for the 67 melanoma patients starting from the cutoff calculated by ROC curves (details are reported in Supplementary Data 6). Results shown as bar plots (Fig. 6F) and Kaplan–Meier curves (Fig. 6G) clearly demonstrated that a higher M2/M1 ratio is a predictor of a worst PFS. In contrast, a lower M2/M1 ratio showed the opposite trend (Log-rank *p* = 0.0472). In summary, deconvolution analyses demonstrated for the first time that a pro-tumoral M2 macrophage transcriptional program is a molecular signature of resistance to MAPKi and is a predictor of response to target therapy in melanoma patients.

### Co-expression of M2 macrophage markers with miRNA-dependent pro-angiogenic/inflammatory factors correlate with worst survival of melanoma patients

**Fig. 7:**
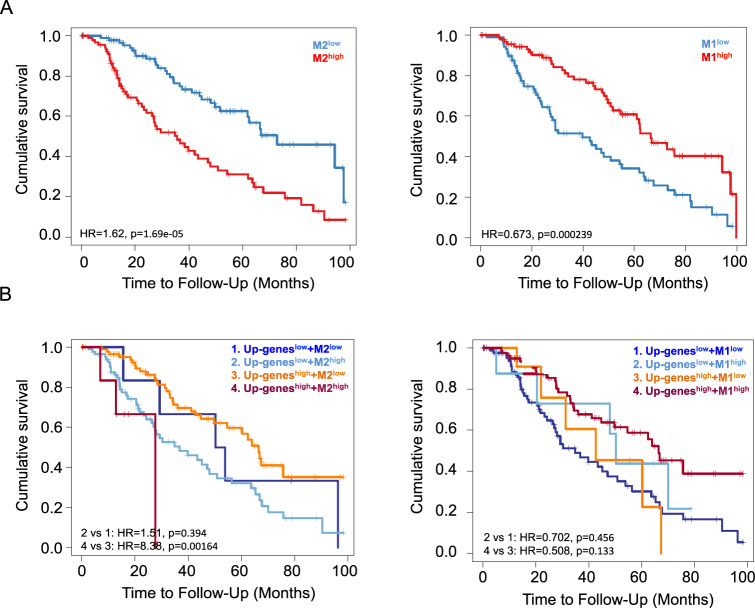
Macrophage signatures and VEGFA, TGFβ1, CCL5 and CXCL2 correlate with survival of melanoma patients based on SKCM data. Kaplan–Meier curves estimating the clinical relevance of M2 (left graphs) or M1 (right graphs) macrophage infiltrates in association with patient clinical outcome alone (**A**) or in combination (**B**) with Up-genes (i.e. VEGFA, TGFβ1, CCL5 and CXCL2). The hazard ratio, p values for Cox models and the log-rank p values are shown on the Kaplan–Meier plots. The hazard ratio, Cox models and the log-rank p values were evaluated to plot KM curves.

Macrophages constitute the most abundant immune cell populations in melanoma and are implicated in tumor progression and metastasis [39]. However, previous studies have failed to demonstrate a prognostic significance of TAM infiltration in melanoma probably because they sought to map macrophage density using single markers such as CD163 and CD68 [40]. Given these premises, we decided to test macrophage-related gene signatures by mining transcriptomic data rather than using single markers. To this aim, we interrogated SKCM data deposited in TCGA (*n* = 471) using TIMER 2.0 software which allows to assess the clinical relevance of immune infiltrates in association with patient clinical outcome [36]. Following this approach, we constructed Kaplan–Meier curves. Our findings (Fig. 7A left panel) show that high M2 macrophage gene signatures (M2^high^) strongly correlate with worse overall survival for melanoma patients. In contrast, M1^high^ melanomas showed the opposite trend according to the anti-tumor potential of these macrophages (Fig. 7A right panel). We previously revealed a positive Spearman correlation of the pro-angiogenic and pro-inflammatory factors represented by our Up-genes and M2 macrophage infiltration in melanomas based on TCGA data (Fig. S9). Hence, we wondered whether the concomitant up-regulation of these gene signatures may have negative prognostic potential for melanoma patients. Kaplan–Meier curves clearly demonstrated that this was indeed the case (Fig. 7B left panel). Notably, melanoma patients whose lesions had Up-genes^high^ + M2^high^ signatures showed worse overall survival as compared to other gene expression combinations (Log Rank *p* = 0.00164). In contrast, when we combined Up-genes with M1 signatures we lost the significant prognostic potential for melanoma patients (Fig. 7B right panel). Overall, these findings have a dual significance: (1) attribute for the first time a prognostic value to macrophage infiltrates in melanoma and (2) demonstrate that M2 macrophages cooperate together with pro-angiogenic and pro-inflammatory factors released by cancer cells and controlled by oncosuppressor miRNAs to shape a TME with a global negative impact for patient survival.

## Discussion

In this paper we provide compelling evidence that LNPs encapsulating selected oncosuppressor miRNAs such as miR-204-5p and miR-199b-5p may be considered as new tools to improve efficacy of current therapies for BRAF mutant melanoma. Interestingly, the enforced expression of these miRNAs is not able to affect the proliferation of BRAF-wt melanoma cells either in the absence or in the presence of NRAS mutations, thus suggesting their specificity only for BRAF-mutated melanomas (Fig. S10A). We show that LNP-miRs are capable of simultaneously hitting many intrinsic and extrinsic oncogenic pathways adopted by melanoma cells to survive BRAF and MEK inhibition. A peculiar aspect of our study relies on the demonstration of the capability of therapeutic LNPs to limit macrophage recruitment into tumors to enhance the growth inhibitory effects of MAPKi. We are also aware that a limitation is the use of partially immunodeficient mouse models that still have macrophages but lack of other immune cells, like all the classes of lymphocytes [41]. Given the impact on TME, our data support transition of RNA therapeutics as tools to improve the efficacy of both MAPKi and ICI therapies to clinical trials. In this context, in stage IV melanoma, five year overall survival rates obtained with the combination of dabrafenib plus tramentinib [2] or ipilimumab plus nivolumab [42] are currently up to 34 and 52%, respectively. Three different Phase III combination studies in the subset of patients harboring BRAF-V600 mutations using a BRAF plus a MEK inhibitor and immunotherapy with anti-PD-1 or anti-PD-L1 have started [43–45]. Although the long-term outcomes are not yet available, the updated results from these ongoing studies demonstrate that: (1) in IMspire150, PFS was increased in the combo therapy arm of ICI + MAPKi vs. MAPKi alone [44], (2) in KEYNOTE-022 the benefits of ICI + MAPKi were mitigated by severe adverse effects [45] whereas (3) in COMBI-i no PFS improvement was observed [43]. However, these different MAPKi+ICI approaches demonstrated to not be able to improve the efficacy of ipilimumab+nivolumab but rather limited by higher toxicity for patients as compared to ICIs. Therefore, the issue still remaining is that a significant proportion of melanoma patients do not benefit from existing therapies both alone or in combination. Based on the data presented in this study, we envision a scenario in which this large proportion of patients may benefit from the combinatorial treatments of therapeutic LNPs plus MAPKi and/or ICIs relying on their capability to hamper the development of an immunosuppressive TME. This hypothesis needs to be tested using fully immunocompetent in vivo models recapitulating an intact murine and/or human immune systems. More importantly, we believe the transition of miRNA-based therapeutics to the clinical practice in oncology will be facilitated by the success in the fight against the SARS-CoV2 pandemic, of LNPs-based RNA vaccines. In recent years many efforts have been made towards the clinical application of miRNA based therapeutics. However at the moment the outcomes of the trials have so far been ambivalent. Indeed, some studies have reported promising results, whereas others have demonstrated limited efficacy and/or toxicity [46]. For example, a miR-34 mimic, namely MRX34, has been tested for its antitumor activity in a multicentre phase I clinical trial in patients with advanced malignancies. However, the trial has been discontinued due to immune-related adverse events, such as cytokine release syndromes [47]. Differently, other trials have produced encouraging results. For example, a tumour-suppressive miR-16 mimic is the object of an ongoing phase I study (o treat patients with non-small cell lung cancer or mesothelioma [48]. Interestingly, a similar replacement therapy using miR-16 mimic has been also proposed to treat melanoma [49], but at the moment this is still in preclinical development. Other encouraging results have been obtained using locked nucleic acid (LNA)-modified antimiRs to inhibit the expression of oncogenic miRNAs [50]. Altogether, these studies suggest that miRNAs can be suitable cancer therapeutics if delivery methods are improved and toxicities are carefully assessed.

Our findings go beyond the potential therapeutic implications because they provide novel insights into the role of non-genetic post-transcriptional mechanisms centered on miRNA deregulation at the basis of drug resistance to targeted therapy in metastatic melanoma. Thanks to a combination of bioinformatic and experimental approaches, we demonstrate here that two oncosuppressor miRNAs, namely miR-204-5p and miR-199b-5p, are the main regulators of several core escape pathways to MAPKi therapy. The co-enrichment of five of them, namely epithelial to mesenchymal transition (EMT), degradation of the extracellular matrix, hypoxia, MAPK signaling pathway and cytokine-cytokine receptor interaction had already been described as hallmarks of targeted therapy resistance [4, 17, 27]. However, they had not been previously linked to the downregulation of these two miRNAs. Interestingly, EMT, hypoxia and angiogenesis-related gene signatures are also up-regulated in anti-PD-1 innately resistant melanomas (referred to as IPRES transcriptional signature) [4, 51]. Hence, we postulated that miR-204-5p and miR-199b-5p could be regulators of core escape pathways of MAPKi resistance which mediate cross-resistance to ICI therapy.

Our data support the notion that MAPKi resistant cells rely on the alteration of pro-angiogenic and inflammatory cues potentially able to reprogram a drug resistant tumor microenvironment. This is witnessed by the aberrant production of four soluble factors, i.e. VEGFA, TGFβ1, CCL5 and CXCL2, which are all molecular targets of miR-204-5p and/or miR-199b-5p. While the first two have already been correlated with MAPKi resistance [4, 25], here for the first time, the latter two have been shown to be involved in this phenomenon. In addition, we provide evidence that the shift from tumor suppressive M1 macrophages to pro-tumoral M2 macrophages is correlated with cancer progression and therapy resistance. Consistently, we demonstrate that MAPKi resistant melanoma cells are able to recruit and re-educate M2 polarized macrophages. More importantly, we confirm that pro-tumoral macrophage transcriptional programs are molecular signatures of MAPKi resistance and predict therapy response in melanoma patients.

Focusing on nanoparticle delivery, we demonstrate that LNPs encapsulating oncosuppressor miRNAs are able to exert both a direct and indirect inhibitory function on melanoma growth in vitro and in vivo. As to the first, they inhibit cell growth if administered alone or in combination with MAPKi [26]. Regarding the indirect functions, oncosuppressor miRNAs are able to hamper the development of a drug resistant TME by re-educating M2 polarized macrophages instructed by drug resistant melanoma cells. Interestingly our in vivo findings suggest that these functions are exerted with different magnitudes between the two cellular models tested. Indeed, in A375-derived tumors the indirect effects on TME dominate over the direct ones. This is witnessed by the strong reduction of a) TAMs infiltrating tumors and b) CD31 positive vessels in LNP-miRs+MAPKi treated mice whereas, in contrast, no effects have been observed on proliferative markers like ki67. Differently, data obtained on M14-derived tumors suggest that the direct and indirect inhibitory functions are in balance. The effects on TME are explained by the inhibitory effects that miR-204-5p and miR-199b-5p have on the production and release of multiple pro-angiogenic and pro-inflammatory factors.Consistently, VEGFA and CCL5 have been described to be recruiter of angiogenic macrophages, whereas TGFβ1 and CXCL2 are able to induce their reprogramming toward a M2 phenotype [35, 52–54]. Given that tumor angiogenesis has been studied for decades, VEGFA clinical targeting led to several FDA-approved drugs. In particular, the humanized anti-VEGFA monoclonal antibody (mAb), i.e. Bevacizumab, is currently used in hundreds clinical trials [55], including in combination with ICI therapies [56]. An example of this is seen in the ongoing Phase II Trial of anti-PD-L1 Atezolizumab + Bevacizumab in unresectable or metastatic melanoma (NCT04356729) for which long-term outcomes are not yet available. Along the same line, a mAb targeting TGFβ1 receptor, namely Fresolimumab showed promising results in a phase I trial involving patients with melanoma [57]. Like in the case of VEGFA, the inhibition of TGFβ signaling is currently under evaluation in multiple clinical trials to enhance the efficacy of cancer immunotherapies [58]. In contrast to VEGFA and TGFβ1, the clinical targeting of CCL5 and CXCL2 in oncology is still at the beginning and, thereby, no efficacy results are currently available [59].

Besides the demonstration of the capability of LNP-miRs in combination with MAPKi to delay or impair the emergence of de novo drug resistance, another challenging field of study is the overcoming of acquired resistance to BRAF and MEK inhibitors in melanoma. This will be investigated in the future using the appropriate cellular models (i.e. A375 DR cells).

One question still remains open regarding the molecular mechanisms driving miR-204-5p and miR-199b-5p downregulation during the development of MAPKi resistance in melanoma. miR-204-5p is one of the best studied miRNAs in melanoma progression and therapy resistance [25, 60–62]. However, it is important to point out that the biological role of this miRNA in the development of resistance to BRAFi has been challenged by some contradictory results depicting it as either an antagonist or facilitator of resistance. We believe that the body of evidence gathered by our group in the last few years answers these contradictions, strengthening the notion of miR-204-5p’s role in antagonizing MAPKi resistance in melanoma. From a molecular point of view, it has been demonstrated that miR-204-5p is negatively regulated by BRAF-V600 driven activation of ERK pathway through MITF and STAT3 transcription factors [62, 63]. This suggests that the reactivation of the MAPK pathway occurring in the vast majority of relapsing melanomas may drive the downregulation of the oncosuppressive miR-204-5p to consolidate the drug-resistant status.

miR-199b-5p has been the object of less studies as compared to miR-204-5p in melanoma. However interestingly enough, it has been reported that in chondrosarcomas miR-199 family members are negatively regulated by an autocrine feedback loop involving CCL5-VEGFA [32]. The data presented here support the hypothesis that the same regulatory network may occur in MAPKi-resistant melanoma cells. Interestingly, by interrogating TCGA data we have unveiled a negative Pearson correlation between the expression levels of miR-204-5p/miR-199b-5p and MITF in melanoma. These bioinformatics analyses suggest that the master transcription factor of melanocyte may be a negative regulator of both these oncosuppressive miRNAs. However, this hypothesis warrants of further experimental validations (Fig. S10B).

Overall, we believe that our findings are of importance (1) at a translational level through the demonstration of in vivo efficacy of LNPs encapsulating oncosuppressor miRNAs for the therapy of metastatic melanoma. and (2) at a mechanistic level because they help to uncover how miRNA deregulation orchestrates the development of drug resistance. A last innovative aspect of our work is represented by the evidence identifying M2 transcriptional programs as molecular signatures of MAPKi resistance able to predict therapy response. This has profound medical implications in the attempt to unravel melanoma immune landscape driving therapy resistance to identify novel biomarkers able to guide clinical decisions.

## Materials and methods

### Cell lines and treatments

All cell lines were routinely tested for mycoplasma and authenticated using Short Tandem Repeat (STR) analysis by the ATCC Cell Line Authentication Service (ATCC, Manassas, VA, USA). All sensitive and MAPKi-resistant human melanoma cell lines used in this study were obtained and cultured as previously described [24, 25]. Briefly, BRAF-mutant M14 and A375 cells have been exposed to increasing concentrations of a BRAFi, i.e. Dabrafenib from 50 nM to 2 μM every two weeks for a total period of 2 months. The effective aquisition of resistance has been tested by proliferation assays using sensitive counterparts as controls. For M15 the IC50 relative to BRAFi are: 169 nM for sens and 1.5 μM for res. For A375 the IC50 relative to BRAFi are: 1.39 nM for sens and 500 nM for res. A375DR cells have been selected in the presence of both BRAF and MEK inhibitors as describe above. The MEKi (i.e. Trametinib) was added to cells at half concentration of the BRAFi. For A375 sens the IC50 are: 1.39 nM+0.9 nM for BRAFi and MEKi, respectively whereas for DR cells are: 500 nM + 250 nM for BRAFi and MEKi, respectively. Human embryonic kidney 293 cells (HEK293) were purchased from System Bioscience (Palo Alto, CA, USA) and cultured according to the manufacturer’s instructions. Human THP-1 monocytes were cultured in RPMI 1640 medium (Euroclone, Milan, Italy) supplemented with 10% inactivated fetal bovine serum (Gibco, Thermo Fisher, Waltham, MA, USA), 1% L-Glutamine and 100 μg/ml penicillin/streptomycin (Euroclone). Dabrafenib and trametinib as BRAFi and MEKi, respectively, were obtained by Novartis Farma S.p.A. (Rome, Italy). Treatments with LNPs were performed by exposing cells to 30 μg of each LNPs in the presence of FBS as previously reported [26]. Viable melanoma cells were determined through CellTiter-Glo® Luminescent Cell Viability (Promega, Madison, WI, USA). For luciferase assays, the plasmids containing the 3′UTR relative to CCL5 (SC210875) or CXCL2 (SC209527) have been purchased by Origene (Rockville, MD, USA) and evaluated by Dual-Luciferase® Reporter Assay System (Promega). For Western blot analyses Phospho-ERK 1/2 (#9101) and Phospho-AKT (#9271) were purchased from Cell Signaling Technology (Danvers, Massachusetts, USA) whereas GAPDH (Sc-32233) was obtained from Santa Cruz Biotechnology (Dallas, Texas, USA).

### Materials, preparation and characterization of LNPs

1,2-dioleyl-3-dimethylammonium propane (DODAP) and N-palmitoyl-sphingosine-1-{succinyl[methoxy(polyethylene glycol)2000]} (PEG2000-Cer16) were purchased by Avanti Polar Lipids. Disteroylphosphatidylcholine (DSPC) was kindly offered from Lipoid GmbH (Cam, Switzerland). Cholesterol (CHOL), sodium chloride, sodium phosphate, HEPES, citric acid and sodium citrate were purchased by Sigma Aldrich (USA). Ethanol and other solvents were obtained by Exacta Optech (Italy). Lipid nanoparticles encapsulating miRNA sequences were prepared by the ethanol injection method followed by extrusion [26]. Briefly, an ethanol lipid stock solution (DSPC/CHOL/DODAP/PEG2000-Cer16-25/45/20/10 w/w) was mixed with a miRNA solution (20 mM citric acid, pH 4) consisting of scrambled miR-204-5p or/and miR-199b-5p sequences at 65 °C. Then, suspension (0.2 mg/mg lipids) was extruded through 200 and 100 nm polycarbonate filters using a thermobarrel extruder (Northern Lipids Inc., Vancouver, BC, Canada) and dialyzed (3,5 kDa cutoff) against citrate buffer (20 mM, pH 4.0) and then HBS (20 mM HEPES, 145 mM NaCl, pH 7.4) to remove excess ethanol and citrate buffer and neutralize the LNP surface. Finally, the amount of non-encapsulated miRNA in LNPs was removed by ultracentrifugation (Optima Max E, Beckman Coulter, USA; rotor TLA 120.2). The size, particles size distribution (PI) and zeta potential (ZP) of LNP formulations were measured by dynamic light scattering with Zetasizer Ultra (Malvern Instruments, Worcestershire, UK) after sample dilution 1:100 v/v with 0.22 μm filtered water. Results were obtained by the average of the measurements of the three different batches of the same formulation. Therefore, the amount of encapsulated miRNA in the LNP formulations was measured after LNP was dissolved in methanol (1:100 v/v) and samples were centrifuged (for 30 min at 13000 rpm; MIKRO 20; Hettich, Tuttlingen, Germany). Supernatants were then analyzed by spectrophotometer at 260 nm. miRNA encapsulation efficiency (EE%) was calculated as % ratio between miRNA actual loading (mg of miRNA/mg of total lipids) and miRNA theorical loading in formulation. LNP characteristics are summarized in Table S1.

### RNA-seq and Whole-Exome Sequencing

Total RNA was extracted from matched BRAFi sensitive and resistant melanoma cells (A375 and M14) using Qiazol (Qiagen, Hilden,Germany), purified from DNA contamination through a DNase I (Qiagen) digestion step. Quantity and integrity of the extracted RNA were assessed by a Nanodrop Spectrophotometer (Nanodrop Technologies LCC, Thermofisher) and by an Agilent 2100 Bioanalyzer (Agilent Technologies, Santa Clara, CA, USA), respectively. RNA library preparation, sequencing and subsequent bioinformatic analyses have previously been described. For identifying DEGs, a filter cut-off criterion of | log2FC | > 0.15 was applied and genes with an adjusted *p* value < 0.05 were considered as statistically significant. The biological function of DEGs was identified by a Gene Ontology (GO) analysis using the R package “enrichR” [64]. Whole exome capture libraries were constructed using the Illumina TruSeq DNA Exome kit. Enriched exome libraries were sequenced on the Illumina NextSeq 500. Raw WES data were analyzed via the Illumina Basespace app Enrichment vv. 2.1.1. which is specifically suited for enrichment-based experiments. Variant VCF files were imported in the Variant Interpreter app for germline and somatic-level annotation. Only high-quality variants with an Allele Frequency>5% and a protein damaging consequence were retained.

### Pathway analysis and target prediction

A Gene Set Enrichment Analysis (GSEA software; https://www.gsea-msigdb. org/gsea/index.jsp) was conducted by using the curated gene sets of the Molecular Signature Database (MSigDB) derived from KEGG, Hallmark, Reactome, and Biocarta collections. GSEA was run in preranked mode using classic as metric and 1000 permutations (FDR < 0.1, *p* value < 0.05, logfold > 0.3). miR-204-5p and miR-199b-5p target genes were predicted by the miRWalk database (http://mirwalk.umm.uni-heidelberg.de/) (binding probability ≥ 0.8).

### RNA extraction and quantitative Real Time PCR (qRT-PCR) analyses

Total RNA was extracted using TRIzol according to the manufacturer’s instruction and quantitated by the Qubit Fluorometer (ThermoFisher Scientific, Foster City, CA, USA). Analyses were performed by the TaqMan Gene Expression Assays for miR-204-5p, miR-199b-5p, U6, VEGFA, TGFβ1, CXCL2, CCL5 and GAPDH. The results were evaluated by the ΔΔCt method. M1, M2, IL6, TNF-α and IL1β genes were tested using the SYBR green dye detection method; the full list of primers used has previously been reported [65]. The mRNA levels were normalized using β-actin [66]. The use of human samples was approved by Istituto Pascale’s Ethical Committee with the protocol DSC/2893 on April 11, 2015. All patients signed a general informed consent, which allowed the use of this material for research purposes and analyzed in an anonymous manner.

### Elisa assays

Soluble VEGFA, TGFβ1, CXCL2 and CCL5 levels from MAPKi sensitive vs. resistant cells and upon LNP treatment were determined by measuring absorbance at 450 nm into a microplate reader using specific ELISA kit according to the manufacturer’s instructions. In particular, VEGFA (#DVE00) was performed by the R&D system (Minneapolis, MN USA), TGFβ1 (#EH0287) and CXCL2 (#EH3178) were carried out by the FineTest (Wuhan, China), CCL5 (#ELH-RANTES-1) was executed by RayBiotech (Peachtree Corners, GA, USA).

### THP-1 differentiation and cell migration assays

THP-1 monocytes were differentiated in macrophages with 100 ng/mL phorbol-12-myristate- 13-acetate (PMA, Sigma-Aldrich, San Louis, USA) for 24 h and then exposed for another 24 h to cell media (CM) deriving from MAPKi sensitive vs. resistant melanoma cells pre-incubated or not with LNPs. For migration experiments, 1 × 10^5^ THP-1 cells were plated in the upper chamber of Transwell (Costar, New York, USA) containing 5 μm pore polycarbonate membrane. As stated before, CM collected was then added in the lower chamber. After 3 h, cells remaining on the top side of the membrane were removed and migrating cells were fixed, stained (Differential Quick Stain Kit, Dade Behring, Marburg, Germany), photographed by using light microscopy, and quantified by counting the number of migrated cells in 10 images for each condition. In all experiments, the CM used for stimulating THP-1 was normalized to the number of adherent cells as previously reported [65].

### Analyses of tumor-infiltrating macrophages and evaluation of clinical outcomes

TIMER2.0 (http://timer.cistrome.org/) was interrogated to estimate the levels of immune infiltration levels for The Cancer Genome Atlas (TCGA) using six state-of-the-art algorithms. Furthermore, this free online software also provides four modules for investigating the link between immune infiltrates, gene expression and clinical outcomes exploring cancer-related associations in the TCGA cohorts. Given the presence of available transcriptome studies in GEO datasets (see appropriate section), we subjected these data to immune infiltration estimation using sample expression matrix in “txt” files formatted with standard delimiters [36].

### CIBERSORTx deconvolution analysis, ROC and Kaplan–Meier curves

Bulk RNA-seq data available in the GEO database (accession numbers are reported below) were subjected to the CIBERSORTx algorithm (available in TIMER2.0) to determine the proportion of 22 different immune cell types in each sample. Absolute values relative to M1 or M2 signatures were then used to create Violin Plots (http://shiny.chemgrid.org/boxplotr/). Starting from these data, we calculated the relative ratio of M2 vs M1 transcriptional programs to plot ROC curves /(http://www.rocplot.org/custom-data/index) [67]. Cut-off values identified by the ROC curves were used to split M2/M1^high^ vs. M2/M1^low^ subgroups shown as Kaplan–Meier models (GraphPad Prism v8.0). Responders (R) vs. Non Responders (NR) were defined following the criteria used in each of the clinical trials, i.e. for GSE50509, GSE65185 and GSE99898 a PFS > 7 months [68]; whereas for GSE77940 and GSE75299 based on Response Evaluation RECIST Criteria for solid tumors [27, 69]. The hazard ratio, Cox models and the log-rank p values were evaluated to plot KM curves.

### Bioinformatic analysis of microRNAs on TCGA data

The correlations of miR-204-5p and miR-199b-5p with melanoma development, prognosis and MITF expression levels have been performed by bioinformatic interrogation of TCGA data. To this purpose, we analyzed miRNA expression levels in a large cohort of 96 Primary melanomas and 350 metastatic ones by employing skin cutaneous melanoma dataset. Normalized and log2-trasformed RPM and RSEM signals of miRNA sequencing data were downloaded from the Firehose Broad GDAC (https://gdac.broadinstitute.org) of the Broad Institute. We used unpaired Student’s T-test and Wilcoxon sign-rank test to assess differences in the miRNAome expression levels when comparing primary and metastatic tumors. The hazard ratio, Cox models and the log-rank p values were evaluated to plot KM curves.

### In vivo experiments

Preclinical studies were performed by subcutaneously injecting 2.5×10^6^ of A375 cells or 5×10^6^ of M14 cells into 6–8-week-old immunodeficient athymic CD1 nude mice. When tumors reached 100 mm [3] mice were randomized and subjected to LNP-Scr, LNP-miR and/or MAPKi (as Dabrafenib+Trametinib). Set up experiments were performed using A375 xenografts and LNPs were injected via tail vein at the dose of 20 μg or 40 μg of LNP-Scr or LNP-miRs. For combinatorial experiments, mice were treated with LNPs (40ug every 72 h i.v.) and/or Dabrafenib+Trametinib (5days/week o.g). BRAFi and MEKi were used at the dose of 10 mg/kg+0.5 mg/kg, respectively for M14 cells and 5 mg/kg+0.1 mg/kg, respectively for A375 cells. Treatments lasted for four weeks and tumor volumes were measured with a caliper. After sacrifice, tumor masses were subjected to qRT-PCR, Western blot and IHC analyses. All procedures involving animals were authorized by the decree n. 26/2014 of the Italian Ministry of Health, authorization n. 787/2015PR-29/07/2015.

### Immunohistochemical (IHC) analyses

Following mice sacrifice, tumors were fixed in 4% buffered formalin and paraffin embedded. Immunohistochemistry analyses were performed using anti-F4/80 (SP115, ThermoFisher), anti-CD31/DIA-310 (Clone SZ31, Dianova), anti-ki67 and hematoxylin and eosin staining. CD31 positivity was determined as the percentage of neo-vessels while F4/80 was quantified as percentage of positivity over tumor cells.

### Melanoma datasets

We analyzed five published melanoma datasets from the Gene Expression Omnibus (https://www.ncbi.nlm.nih.gov/geo/):

- GSE65185 [13]
- GSE77940 [69]
- GSE50509 [70]
- GSE75299 [27]
- GSE99898 [71]

### Statistical Analysis

In vitro experiments were replicated at least three times, unless otherwise indicated, and the data were expressed as average ±SD or ±SE of the mean (SEM). Statistical analyses were performed using GraphPad Prism v8.0 software. In vitro and in vivo groups were compared by Student’s t test or Wilcoxon Signed Rank Sum Test as indicated and statistical significance is represented as follows: *p < 0.05; **p < 0.01; and ***p < 0.001.

## Supplementary information

Table S1

Supplementary Information

Suppl. Figure 1

Suppl. Figure 2

Suppl. Figure 3

Suppl. Figure 4

Suppl. Figure 5

Suppl. Figure 6

Suppl. Figure 7

Suppl. Figure 8

Suppl. Figure 9

Suppl. Figure 10

Suppl. Data 1 RNA-seq

Suppl. Data 2 WES

Suppl. Data 3 GSEA

Suppl. Data 4 Venn diagram

Suppl. Data 5 CYBERSORTX

Suppl. Data 6 M2:M1

## Data Availability

The public datasets used in this study listed in the appropriate “Methods” section are available on Gene Expression Omnibus (GEO) database. All the other data are available from the corresponding author on reasonable request.
