# Supplementary material for: Oncosuppressive miRNAs loaded in lipid nanoparticles potentiate targeted therapies in BRAF-mutant melanoma by inhibiting core escape pathways of resistance
Source: Oncogene. 2022 Nov 23;42(4):293–307. doi: 10.1038/s41388-022-02547-9 (PMC9684877; doi:10.1038/s41388-022-02547-9)
Supplement: Supplementary file 1 — Table S1 [file 41388_2022_2547_MOESM1_ESM.docx]

| **Formulation** | **Mean**  **(nm) ± SD** | **PI ± SD** | **ZP**  **(mV ± SD)** | **Actual loading**  **(μg miRNA/mg lipids)** | **EE% ± SD** |
| --- | --- | --- | --- | --- | --- |
| LNP-Scr | 137.3 ± 7.2 | 0.12 ± 0.02 | -15.2 ± 3.4 | 186.4 ± 0.004 | 93.2 ± 2.3 |
| LNP-miR-204-5p/199b-5p | 162.2 ± 1.6 | 0.16 ± 0.01 | -27.2 ± 5.4 | 184.4 ± 0.002 | 92.2 ± 3.2 |

**Table S1**. Characteristics of LNP-Scr or encapsulating miR-204-5p/199b-5p.
