# Supplementary material for: Oncosuppressive miRNAs loaded in lipid nanoparticles potentiate targeted therapies in BRAF-mutant melanoma by inhibiting core escape pathways of resistance
Source: Oncogene. 2022 Nov 23;42(4):293–307. doi: 10.1038/s41388-022-02547-9 (PMC9684877; doi:10.1038/s41388-022-02547-9)
Supplement: Supplementary file 2 — Supplementary Information [file 41388_2022_2547_MOESM2_ESM.docx]

**Supplementary Information**

**Figure S1** LNPs delivering oncosuppressor miRs reduce A375-derived tumors *in vivo.* **A** Schematic illustration of the set up studies performed on xenograft models of A375 melanoma cells. **B** Bar plots showing the measurements of the tumor volumes of mice injected with A375 (n=3) and treated LNP-Scr or LNP-miRs. These experiments were performed with LNPs injected via tail vein at the dose of 20 μg or 40 μg of LNP-Scr or LNP-miRs. **C** Quantification of miR-199b-5p (left graph) and miR-204-5p (right graph) in tumors treated as previously reported by using qRT–PCR at different time points (i.e. 24, 48, 72 and 96 hours). **D** Quantification of VEGFA (left graph) and BCL2 (right graph) in tumors by using qRT–PCR as previously stated. *p < 0.05; **p < 0.01; and ***p < 0.001. Results are represented as the mean (n=3) ± SD.

**Figure S2** Weight measure and experimental analyses on tumors derived from mice injected with A375 or M14 cells. **A** Mouse weight determination as a parameter to monitor animal suffering in response to the indicated treatments. **B** Quantification of miR-204-5p and miR-199b-5p expression levels in tumors treated wit LNP-miRs by using qRT–PCR. **C** Western blot analyses have been performed on tumor masses collected after mice sacrifice and tested for the indicated antibodies. GAPDH protein has been used as housekeeping for the equal loading. IHC analyses determining cell proliferation using ki67 marker, the percentage of necrosis and neo-vessel formation through the endothelial cell marker CD31 in A375 (**D**) and M14 (**E**) derived tumors. *p < 0.05; **p < 0.01; and ***p < 0.001. Results are represented as the mean (n=7 for A375; n=10 for M14) ± SEM.

**Figure S3** Whole Exome Sequencing (WES) of A375 cells BRAFi resistant vs. sensitive counterparts. **A** Venn Diagrams showing the genetic variants exclusive or in common between A375-res/sens melanoma cells. **B** The variant allele frequency (VAF) differences of A375 resistant cells vs. sensitive counterparts. Raw WES data were analyzed via the Illumina Basespace app Enrichment vv. 2.1.1. which is specifically suited for enrichment-based experiments. Variant VCF files were imported in the Variant Interpreter app for germline and somatic-level annotation. Only high-quality variants with an Allele Frequency>5% and a protein damaging consequence were retained. (**C**) Western blot analyses have been performed on A375 and M14 melanoma cells sensitive or resistant to BRAFi to measure the activation of pERK and pAKT. GAPDH protein has been used as housekeeping for the equal loading.

**Figure S4** Box plots estimating the correlation of miR-204-5p and miR-199b-5p with melanoma development and prognosis have been performed by bioinformatic interrogation of TCGA data. miRNA expression levels have been tested in a large cohort of 96 Primary melanomas and 350 metastatic ones by employing SKCM dataset and normalized by log2-trasformed RPM and RSEM signals. Unpaired Student’s T-test and Wilcoxon sign-rank test were used to assess differences in the miRNAome expression levels comparing primary and metastatic tumors. The hazard ratio, Cox models and the log-rank p-values were evaluated to plot KM curves.

**Figure S5** VEGFA, TGFβ1, CCL5 and CXCL2 are up-regulated by MAPKi-resistant melanoma cells and are inhibited by LNPs delivering oncosuppressor miRs. **A** Quantification of miR-199b-5p and miR-204-5p by using qRT–PCR in A375 rendered double resistant to both BRAF and MEK inhibitors, i.e. A375DR. **B** Elisa assays measuring VEGFA, TGFβ1, CCL5 and CXCL2 soluble levels in CM coming from A375DR cells. **C** Quantification of miR-199b-5p and miR-204-5p by using qRT–PCR in A375DR cells upon 48 hours of exposure to LNP-Scr or LNP-miRs. **D** Elisa assays measuring VEGFA, TGFβ1, CCL5 and CXCL2 levels in CM coming from A375DR upon LNPs’ exposure. For these experiments cells have been serum starved for 24 hours and then CM have been collected; results were determined by measuring absorbance at 450 nm into a microplate reader. **E** Cell viability evaluation by measuring ATP content in BRAF-mutant WM115 melanoma cells left untreated, treated with Dabrafenib (as BRAFi, 500 nM) in combination with Trametinib (as MEKi, 10 nM) (MAPKi) in the presence of LNP-Scr or LNP-miR (30 μg each). *p < 0.05; **p < 0.01; and ***p < 0.001. qRT-PCR data are represented as mean (n=3) ± SD; Elisa results are expressed as the mean of at least three independent experiments ±SEM.

**Figure S6** Correlation analysis between CCL5, VEGFA, TGFβ1 and CXCL2 expression levels and monocyte infiltration performed on The Cancer Genome Atlas (TCGA) Skin Cutaneous Melanoma (SKCM) data (n=471) through TIMER 2.0 online tool. Tumor purity represented the proportion of cancer cells in a given sample.

**Figure S7** Macrophage infiltration in the A375 and M14 derived tumors. Representative images of IHC analyses by F4/80 antibody determining macrophage infiltration in tumor masses derived from A375 (left panels) and M14 (right panels) in response to the indicated treatments. Original magnification = ×20.

**Figure S8** LNPs delivering oncosuppressor miRs reduce VEGFA, TGFβ1, CCL5 and CXCL2 mRNA levels in BRAFi-resistant cells and in THP-1 monocytes exposed to CM derived from melanoma cells. **A** Quantification of the aforementioned gene expression levels by using qRT–PCR in A375 and M14 res/sens cells (black bars) or in resistant cells treated with LNP-Scr or LNP-miR (green bars). **B** and **C** qRT–PCR of VEGFA, TGFβ1, CCL5, CXCL2, IL6, TNF-α and IL1β in THP-1 cells exposed to CM coming from res/sens melanoma cells (black bars) or CM coming from resistant cells treated with LNP-Scr or LNP-miR (green bars). For these experiments, THP-1 monocytes were differentiated in macrophages with 100 ng/mL phorbol-12-myristate- 13-acetate for 24 hours and then exposed for another 24 hours to cell media (CM) deriving from melanoma cells. *p < 0.05; **p < 0.01; and ***p < 0.001. qRT-PCR data are represented as the mean of at least three independent experiments ± SEM.

**Figure S9** VEGFA, TGFβ1, CCL5 and CXCL2 levels correlate with M2 macrophage infiltration in melanomas. The Cancer Genome Atlas (TCGA) data interrogated through TIMER 2.0 to assess the correlation between the set of upregulated genes with macrophage infiltration in SKCM (n=471). Tumor purity represented the proportion of cancer cells in a given sample.

**Figure S10. A** For these experiments three different melanoma cell lines with a different pattern of oncogene mutations, namely ME1007 (BRAF-wt/NRAS-wt), ME4405 (BRAF-wt/NRAS-mut) and 2/17 (BRAF-wt/NRAS-mut) have been left untreated or treated with LNP-Scr or LNP-miR (30 μg each). After 48 hours, cell viability has been evaluated by measuring ATP content to determine the presence of metabolically active cells. Cell viability results are expressed as the mean of at least three independent experiments ±SEM. **B** The Pearson correlations of miR-199b-5p and miR-204-5p with MITF have been determined by bioinformatic interrogation of TCGA data (SKCM dataset).

**Additional file 9 Table S1** Characteristics of LNP-Scr or encapsulating miR-204-5p/199b-5p

**Suppl. Data 1 RNA-seq analyses**

**Suppl. Data 2 WES analyses**

**Suppl. Data 3 GSEA**

**Suppl. Data 4 Venn diagram**

**Suppl. Data 5 CYBERSORTX analyses**

**Suppl. Data 6 M2/M1 signatures**
