# Supplementary figures and images for: Oncosuppressive miRNAs loaded in lipid nanoparticles potentiate targeted therapies in BRAF-mutant melanoma by inhibiting core escape pathways of resistance
Source: Oncogene. 2022 Nov 23;42(4):293–307. doi: 10.1038/s41388-022-02547-9 (PMC9684877; doi:10.1038/s41388-022-02547-9)

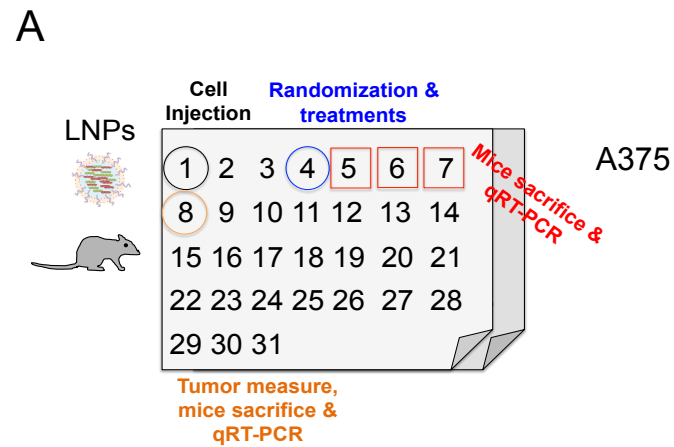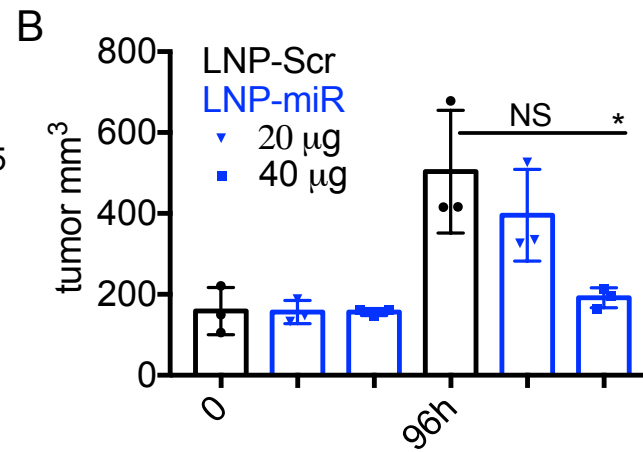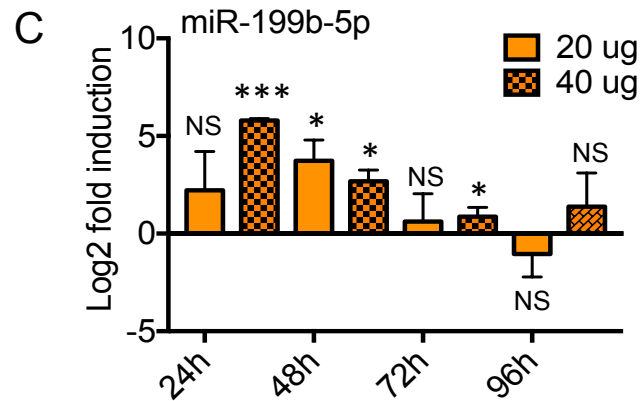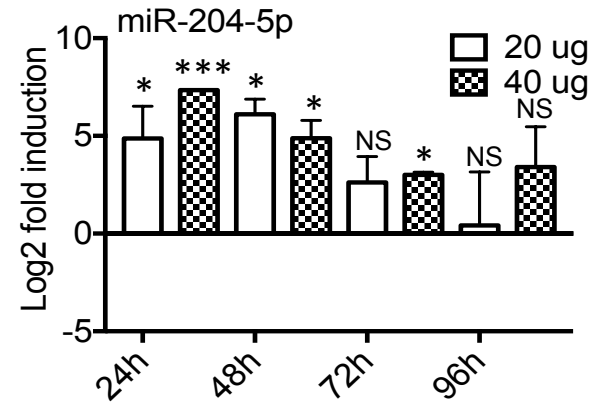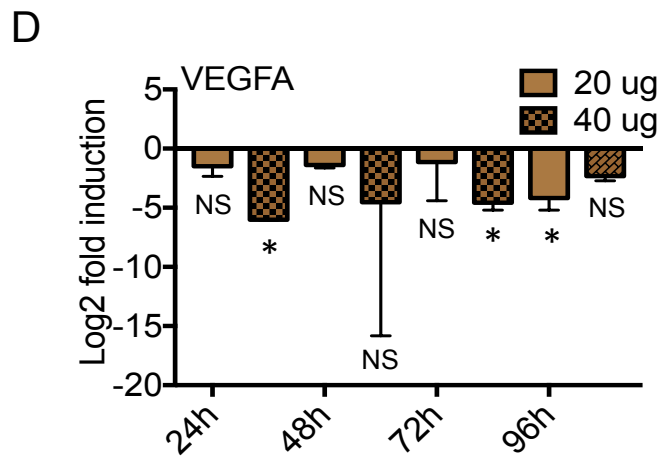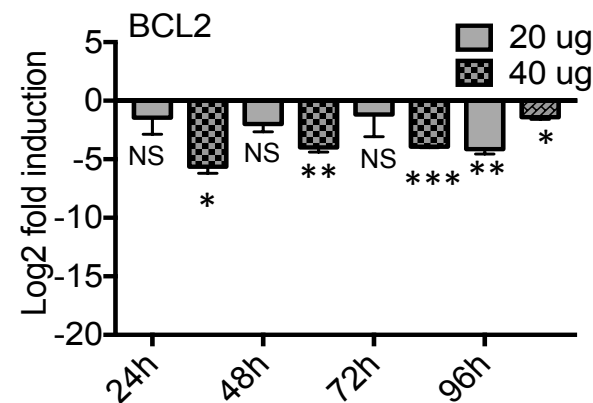

Supplement: Supplementary file 3 — Suppl. Figure 1 [file 41388_2022_2547_MOESM3_ESM.pdf]

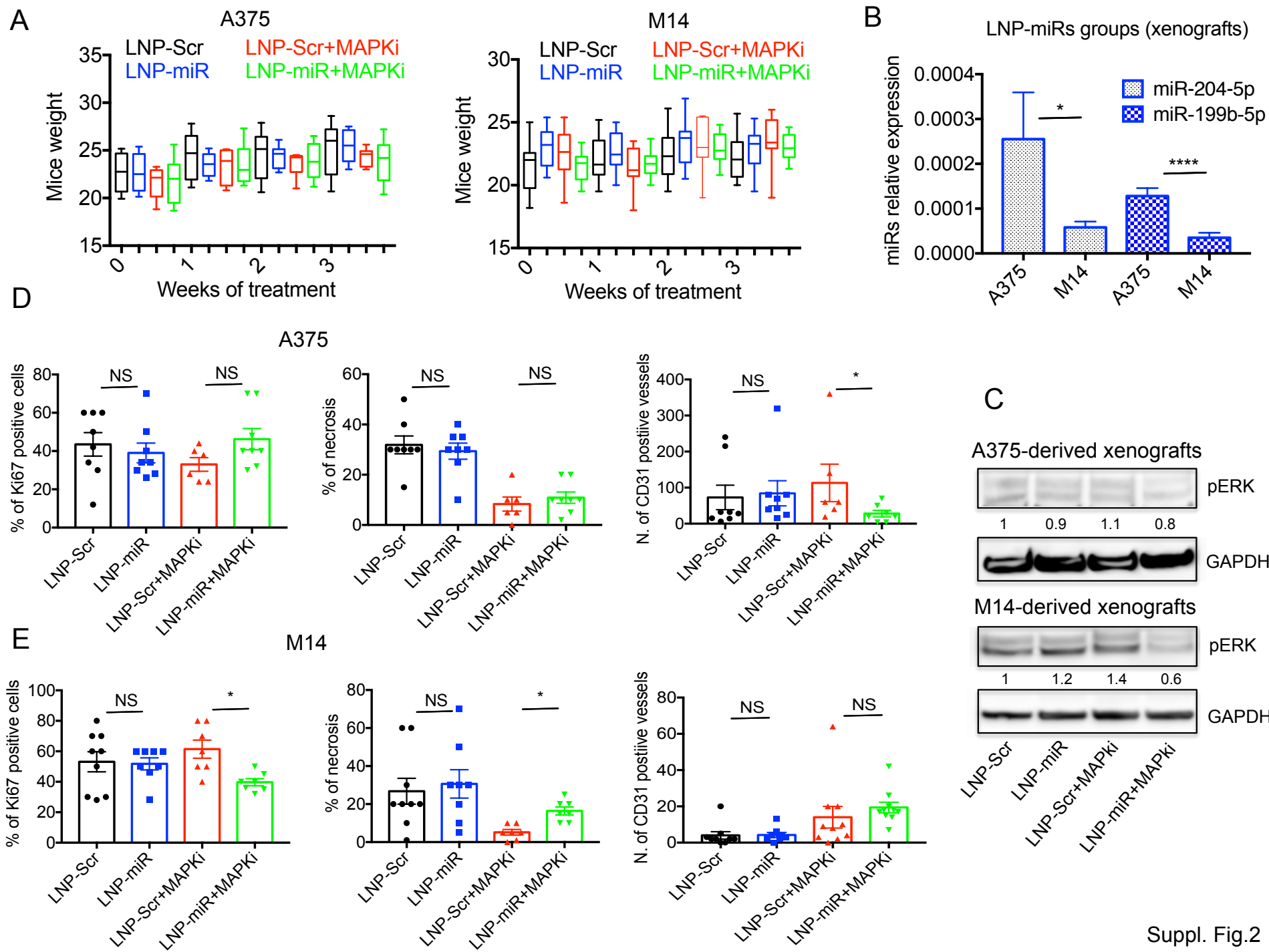

Suppl. Fig.2

Supplement: Supplementary file 4 — Suppl. Figure 2 [file 41388_2022_2547_MOESM4_ESM.pdf]

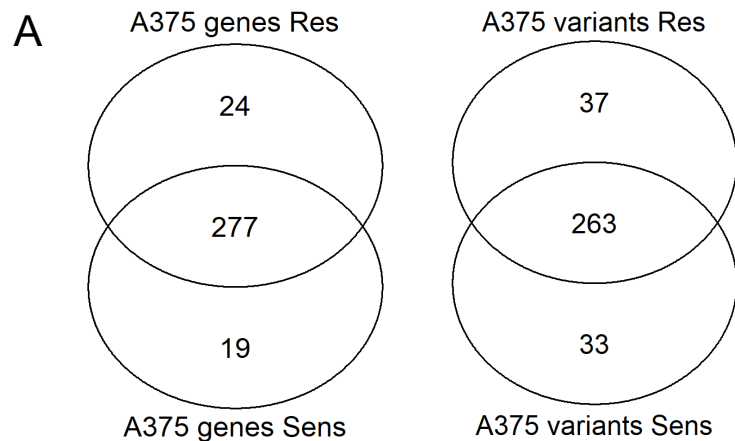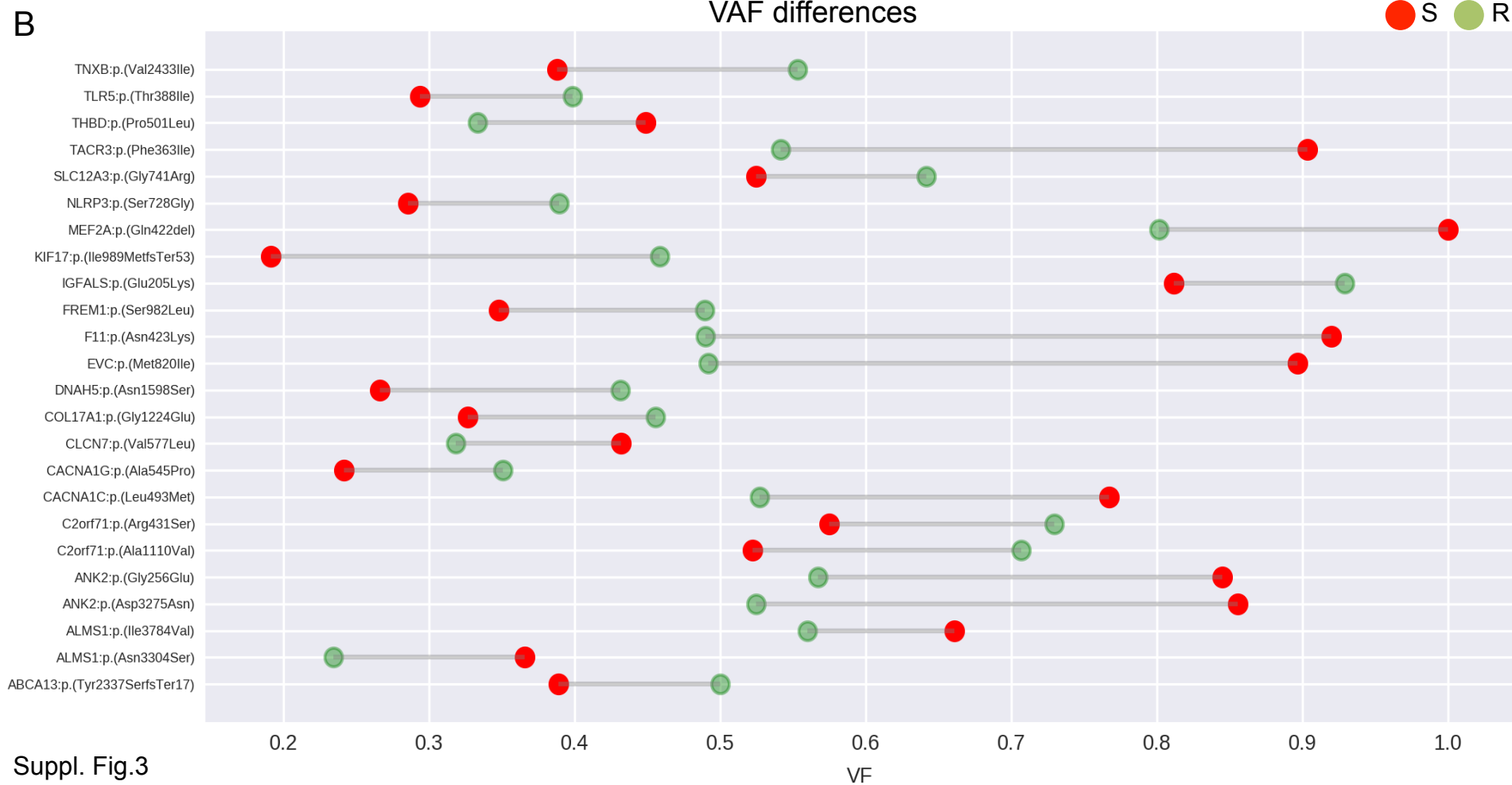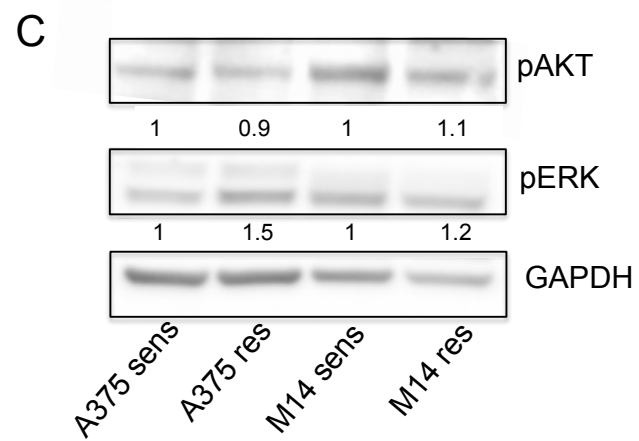

Suppl. Fig.3

Supplement: Supplementary file 5 — Suppl. Figure 3 [file 41388_2022_2547_MOESM5_ESM.pdf]

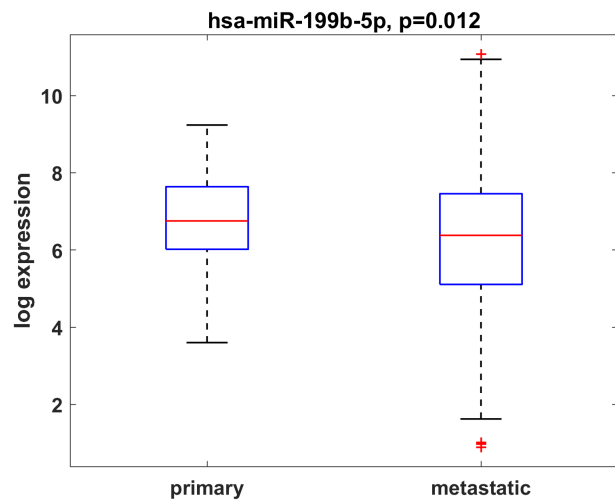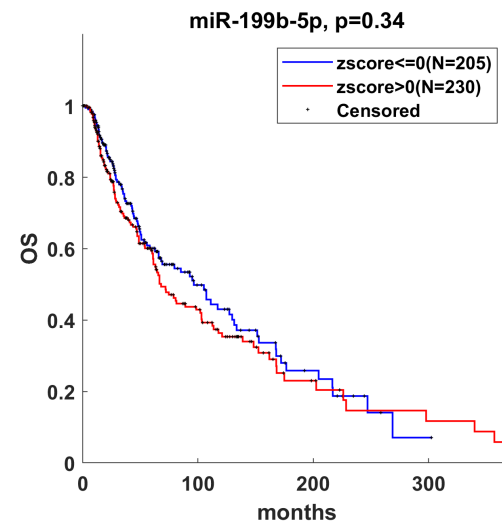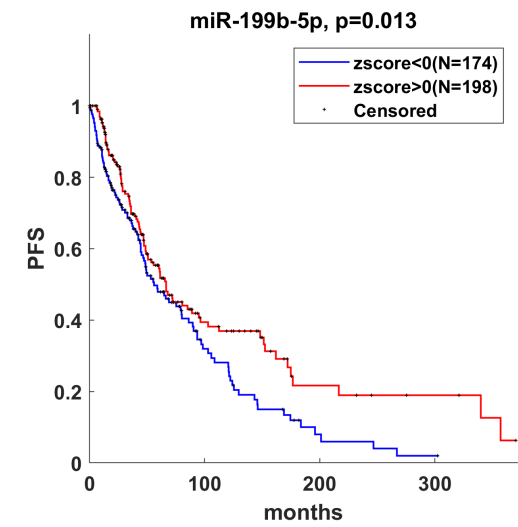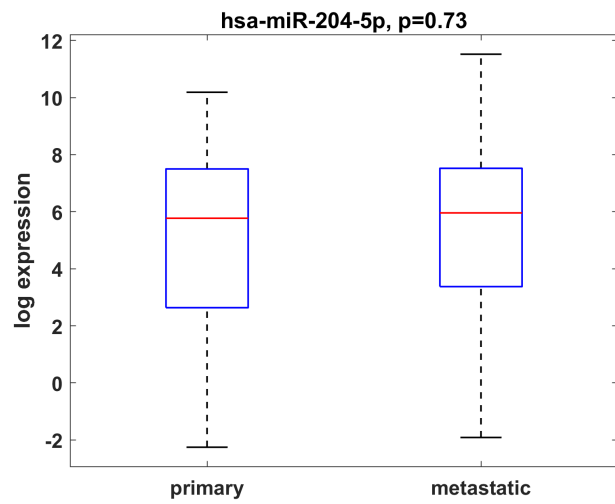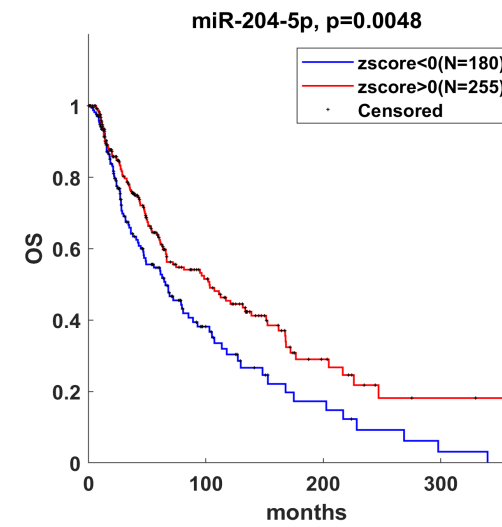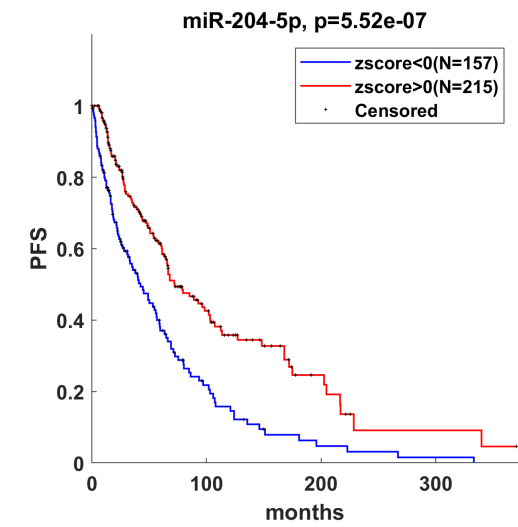

Supplement: Supplementary file 6 — Suppl. Figure 4 [file 41388_2022_2547_MOESM6_ESM.pdf]

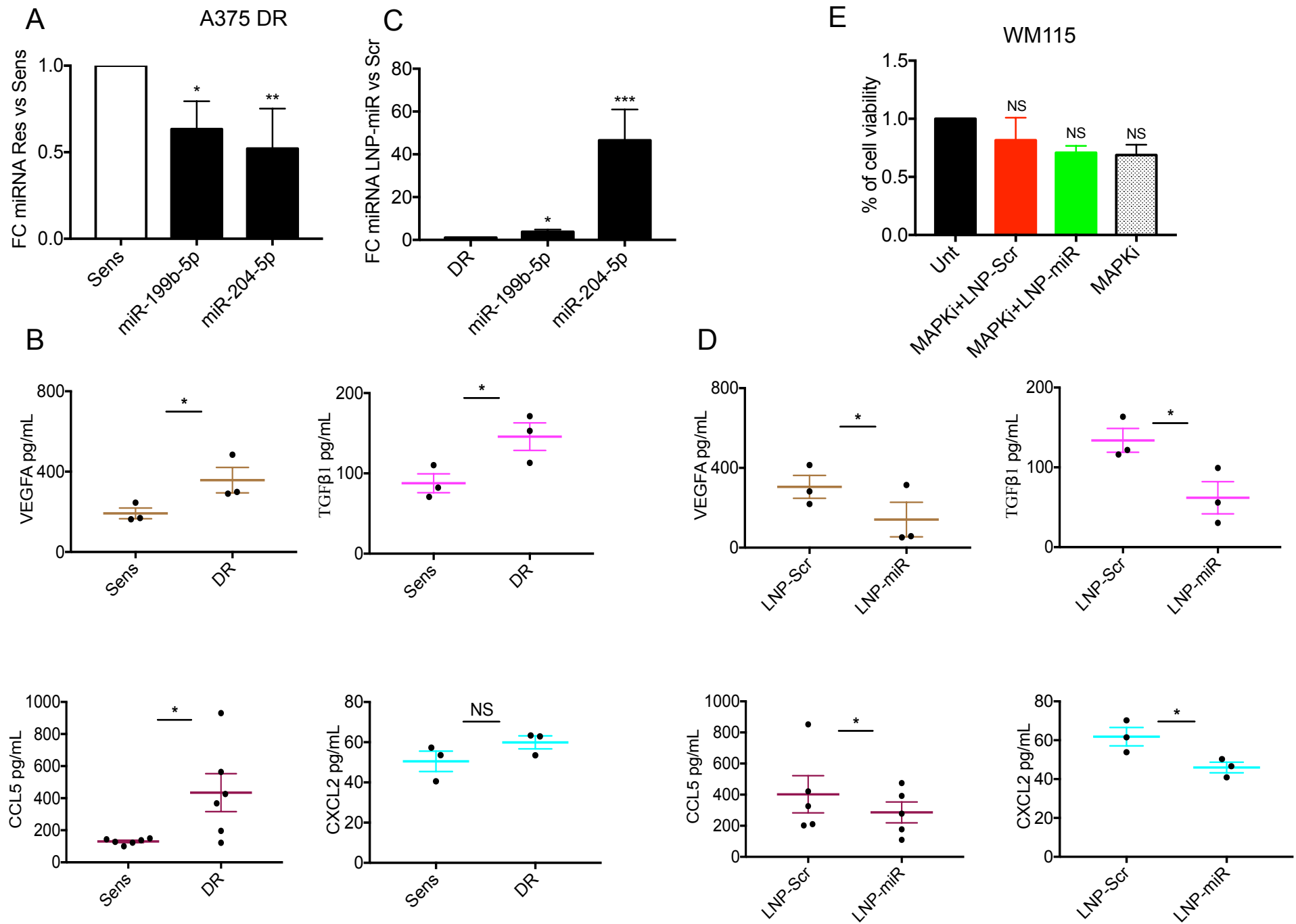

Suppl. Fig.5

Supplement: Supplementary file 7 — Suppl. Figure 5 [file 41388_2022_2547_MOESM7_ESM.pdf]

# SKCM (n=471)

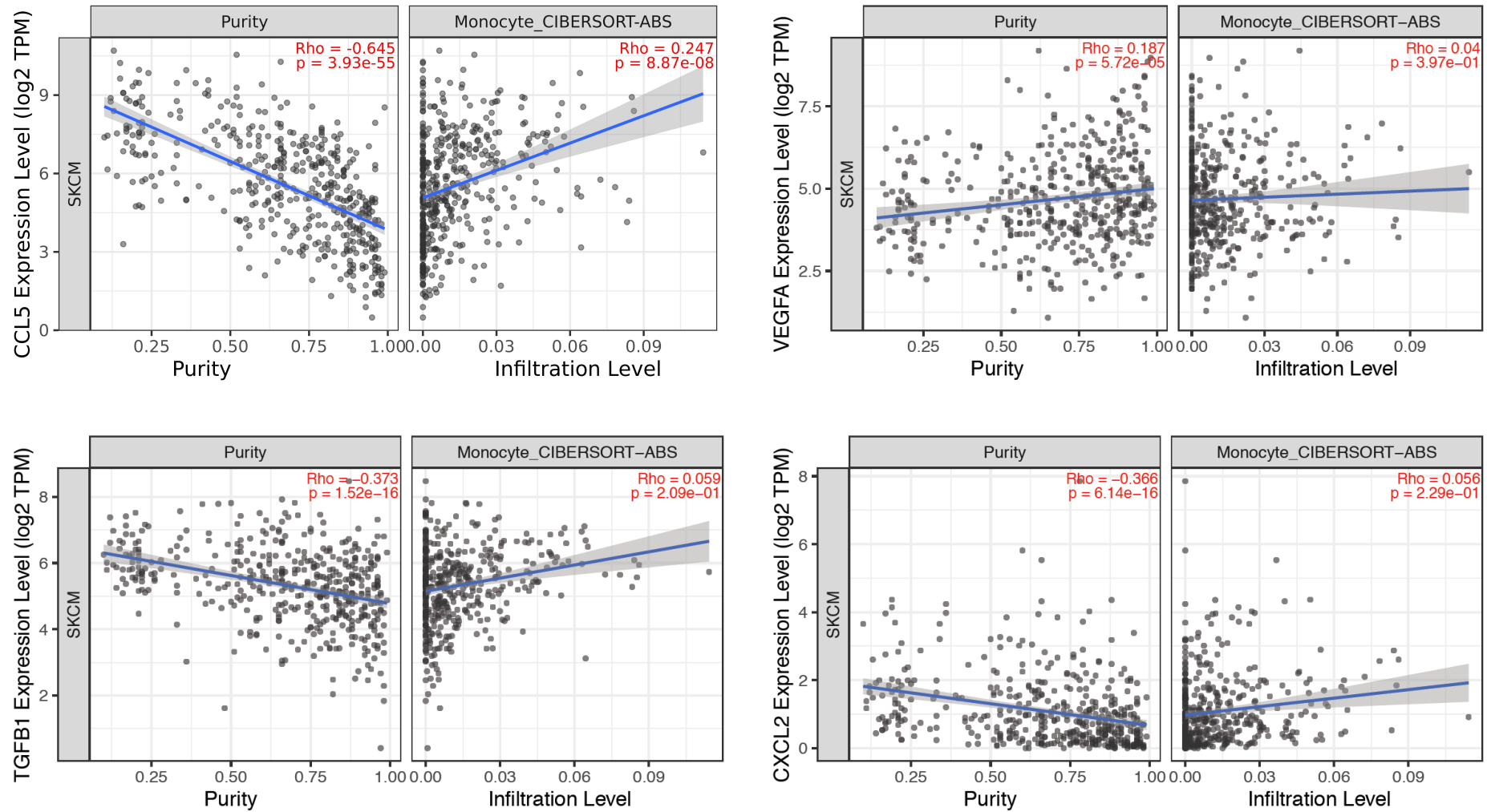

Supplement: Supplementary file 8 — Suppl. Figure 6 [file 41388_2022_2547_MOESM8_ESM.pdf]

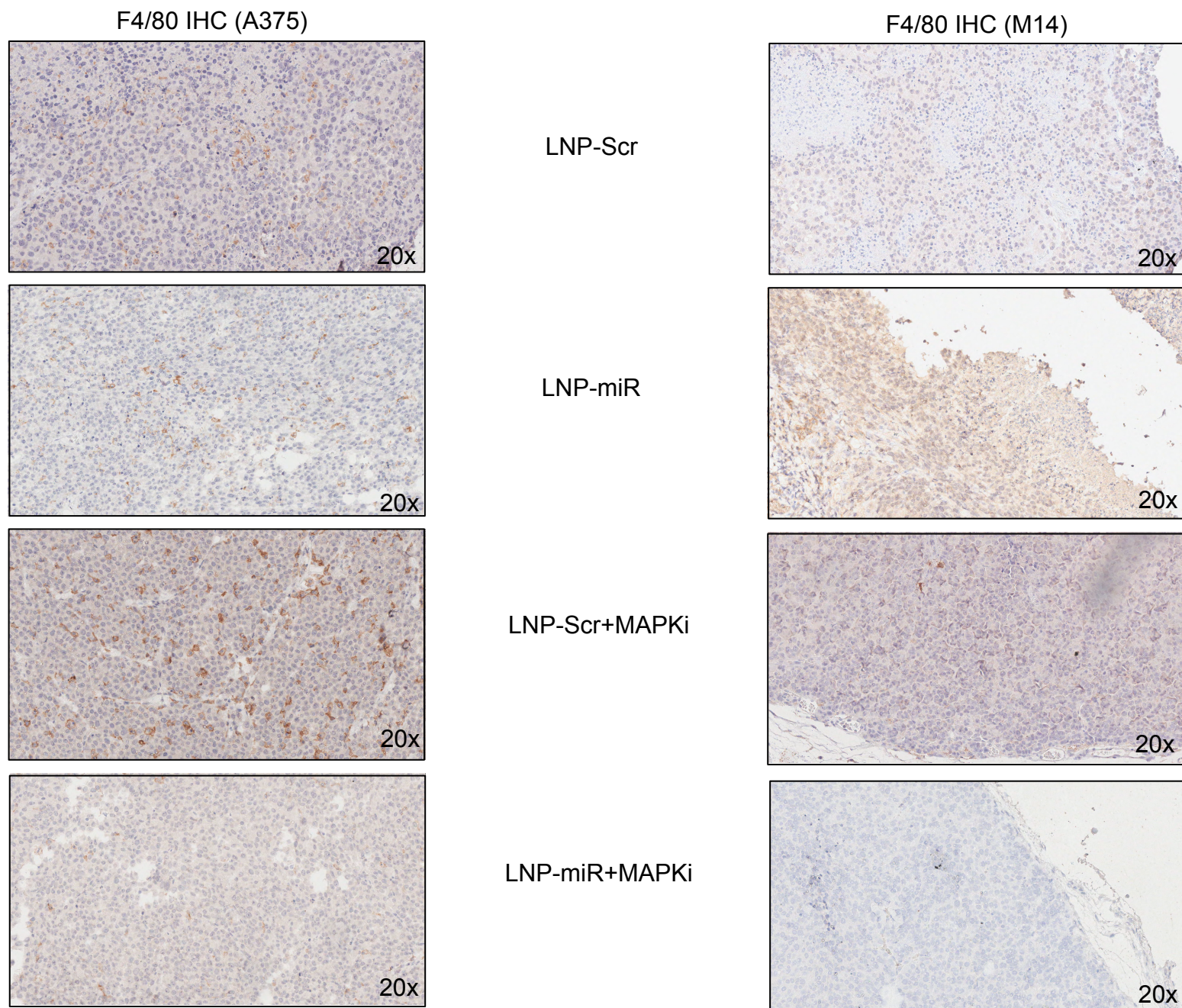

Suppl. Fig.7

Supplement: Supplementary file 9 — Suppl. Figure 7 [file 41388_2022_2547_MOESM9_ESM.pdf]

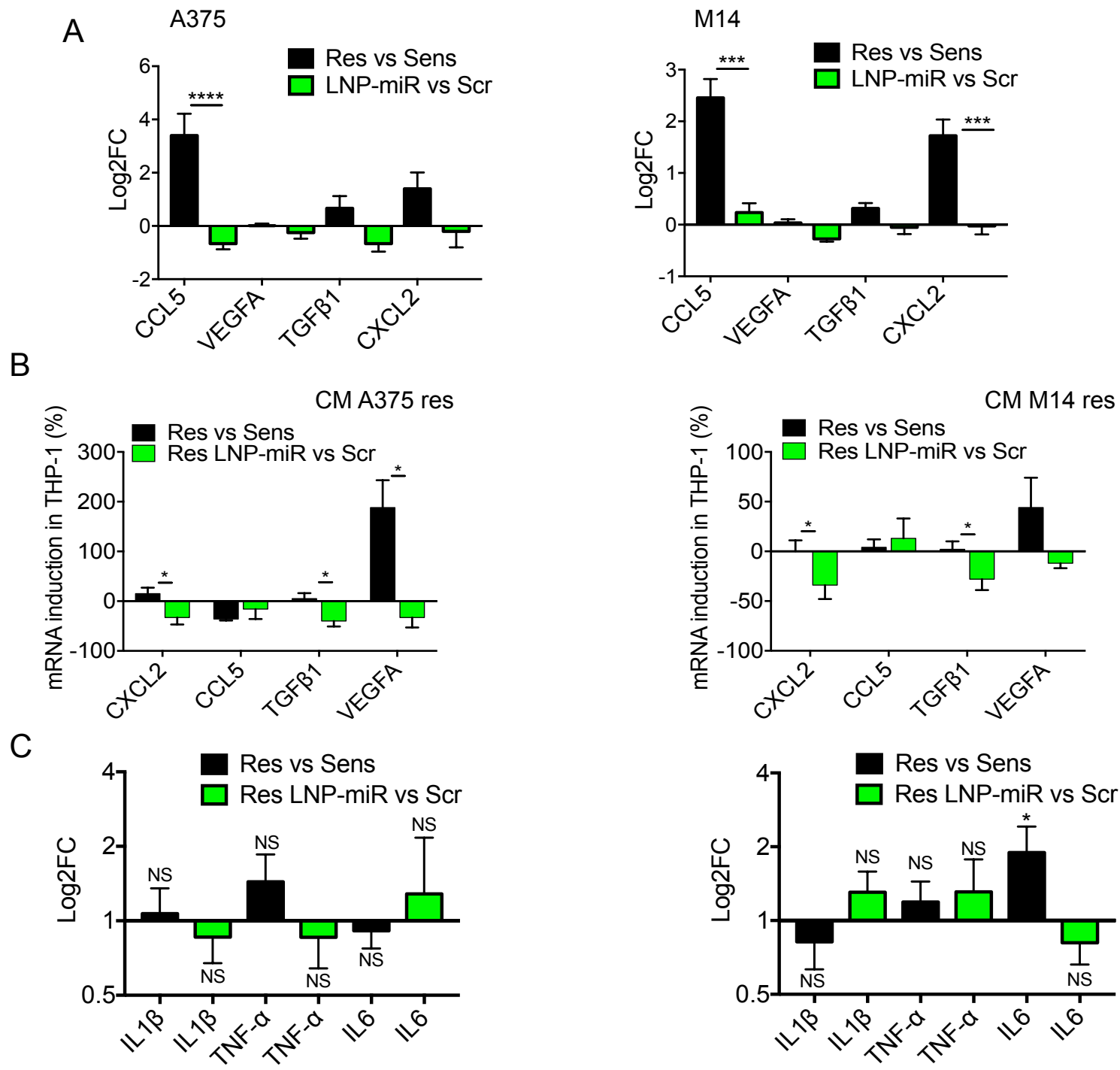

Suppl. Fig.8

Supplement: Supplementary file 10 — Suppl. Figure 8 [file 41388_2022_2547_MOESM10_ESM.pdf]

SKCM (n=471)

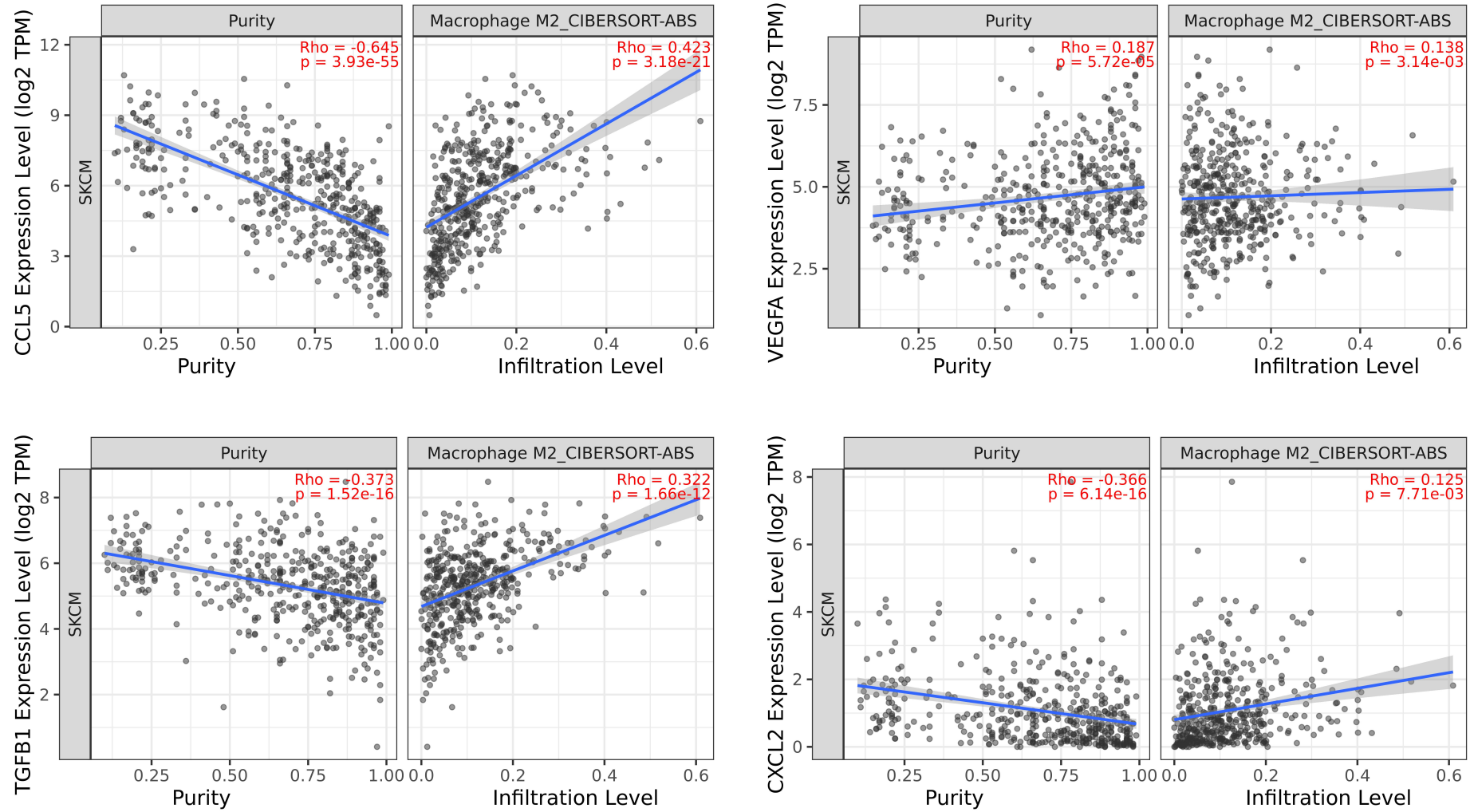

Supplement: Supplementary file 11 — Suppl. Figure 9 [file 41388_2022_2547_MOESM11_ESM.pdf]

A

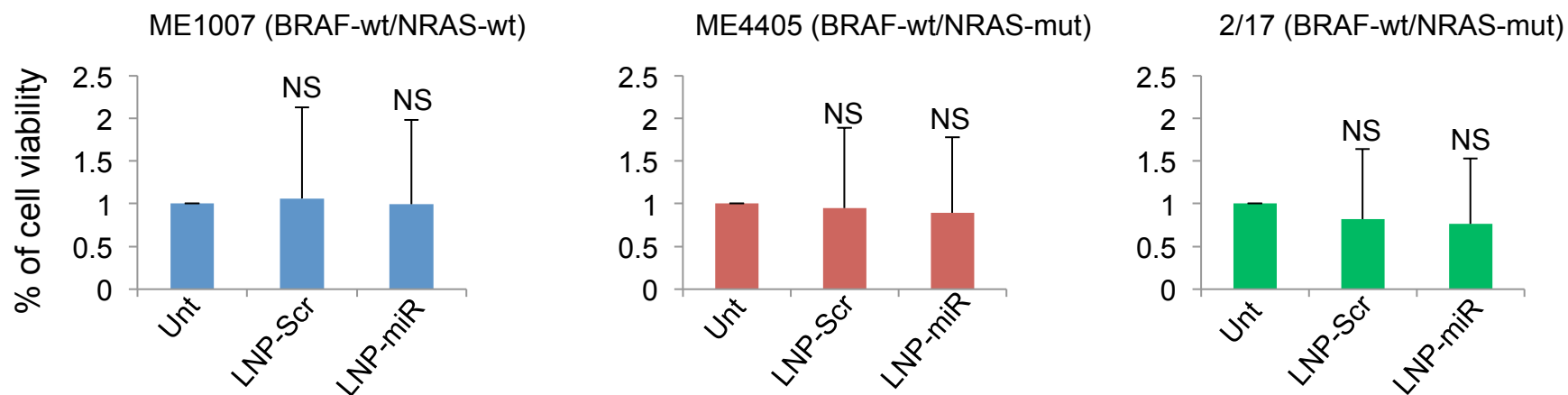

B

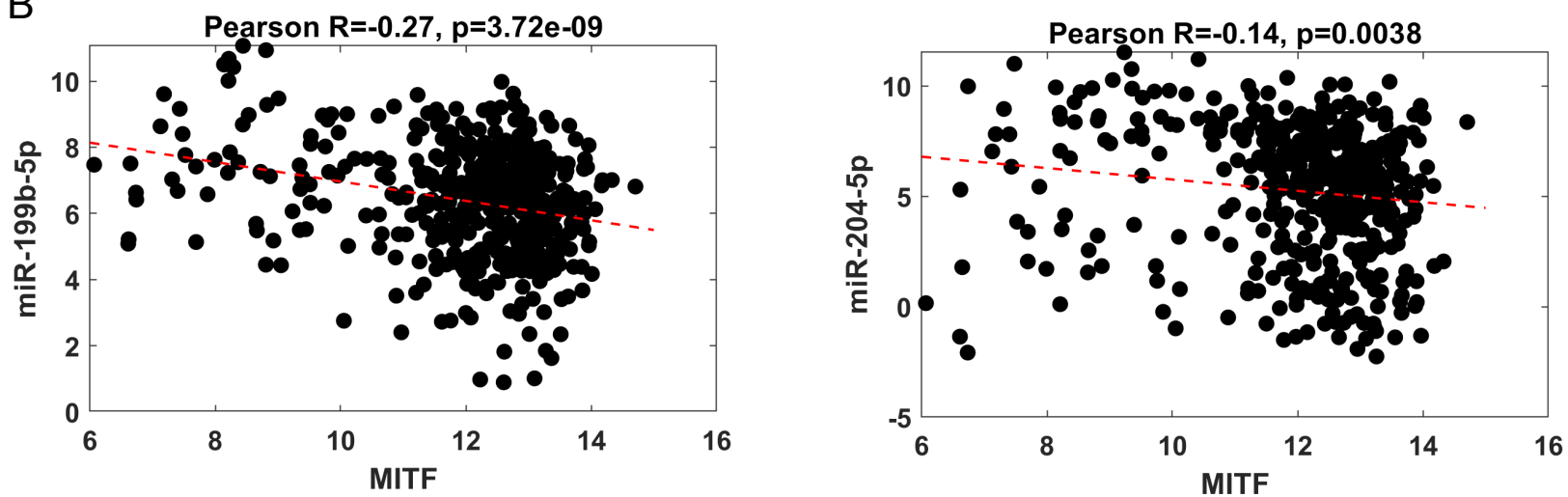

Supplement: Supplementary file 12 — Suppl. Figure 10 [file 41388_2022_2547_MOESM12_ESM.pdf]
